# Evaluating Scalable Supervised Learning for Synthesize-on-Demand Chemical Libraries

**DOI:** 10.1021/acs.jcim.3c00912

**Published:** 2023-08-25

**Authors:** Moayad Alnammi, Shengchao Liu, Spencer S. Ericksen, Gene E. Ananiev, Andrew F. Voter, Song Guo, James L. Keck, F. Michael Hoffmann, Scott A. Wildman, Anthony Gitter

**Affiliations:** † Department of Computer Sciences, University of Wisconsin−Madison, Madison, Wisconsin 53706, United States; ‡ Morgridge Institute for Research, Madison, Wisconsin 53715, United States; § Department of Information and Computer Science, King Fahd University of Petroleum & Minerals, Dhahran 31261, Saudi Arabia; ∥ Small Molecule Screening Facility, University of Wisconsin−Madison, Madison, Wisconsin 53792, United States; ⊥ Department of Biomolecular Chemistry, University of Wisconsin−Madison, Madison, Wisconsin 53706, United States; # McArdle Laboratory for Cancer Research, University of Wisconsin−Madison, Madison, Wisconsin 53705, United States; ¶ Department of Biostatistics and Medical Informatics, University of Wisconsin−Madison, Madison, Wisconsin 53792, United States

## Abstract

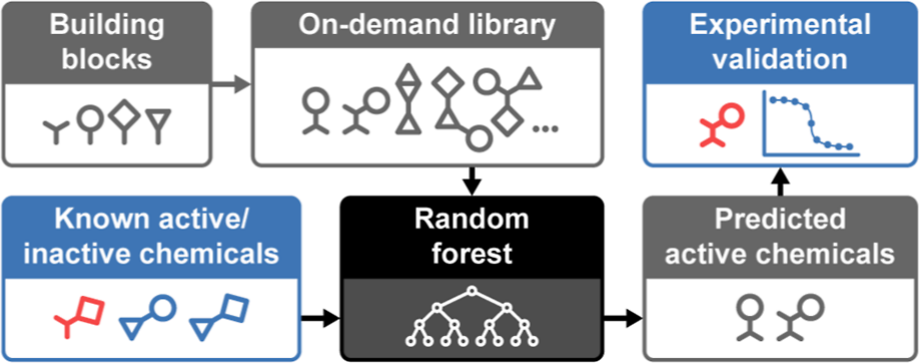

Traditional small-molecule drug discovery is a time-consuming and costly endeavor. High-throughput chemical screening can only assess a tiny fraction of drug-like chemical space. The strong predictive power of modern machine-learning methods for virtual chemical screening enables training models on known active and inactive compounds and extrapolating to much larger chemical libraries. However, there has been limited experimental validation of these methods in practical applications on large commercially available or synthesize-on-demand chemical libraries. Through a prospective evaluation with the bacterial protein–protein interaction PriA-SSB, we demonstrate that ligand-based virtual screening can identify many active compounds in large commercial libraries. We use cross-validation to compare different types of supervised learning models and select a random forest (RF) classifier as the best model for this target. When predicting the activity of more than 8 million compounds from Aldrich Market Select, the RF substantially outperforms a naïve baseline based on chemical structure similarity. 48% of the RF’s 701 selected compounds are active. The RF model easily scales to score one billion compounds from the synthesize-on-demand Enamine REAL database. We tested 68 chemically diverse top predictions from Enamine REAL and observed 31 hits (46%), including one with an IC_50_ value of 1.3 μM.

## 1 Introduction

Access to very large chemical libraries opens new opportunities in drug discovery but presents major scalability challenges for traditional chemical screening workflows. Commercial libraries, which are the primary source of compounds in academic screening efforts, can be grouped into two categories: “in-stock” and “synthesize-on-demand.” As of 2021, in-stock libraries comprise over 12 million molecules previously synthesized and physically stored by vendors.^1^ Synthesize-on-demand libraries are databases containing virtual molecules that a vendor considers to be readily accessible given stocks of available building blocks and established synthetic routes.^2,3^ Such libraries now measure in the billions: the ZINC-22 database aggregates 37 billion compounds from the Enamine REAL, WuXi GalaXi, and Mcule Ultimate catalogues.^4^ Synthesize-on-demand libraries have superior chemical scaffold diversity and coverage of chemical shape characteristics,^1^ unlocking new possibilities to manipulate biological targets. The growth of synthesize-on-demand libraries has been disruptive, requiring new scalable strategies for enumerating, storing, and searching them.^2,3^ However, the question remains as to how to effectively prioritize molecules from synthesize-on-demand libraries for acquisition and testing in the hit identification phase of drug discovery projects.

Virtual screening is essential for guiding the selection of compounds from vast synthesize-on-demand chemical resources. Virtual screening uses computational methods to select compounds to test experimentally against a target of interest.^5−8^ An efficient virtual screening algorithm can exhaustively assess compounds in a very large library so that only the most promising compounds are screened experimentally. Two broad classes of virtual screening approaches, structure-based and ligand-based, have had initial application to large libraries. Structure-based virtual screening, which includes docking,^9,10^ uses the three-dimensional shape of the target protein structure to evaluate candidate ligands. Despite recent examples that successfully use docking to filter 10^8^ to 10^10^ compounds down to hundreds of interesting compounds,^11−13^ there are inherent drawbacks to this approach. Structure-based screening is limited to targets having reasonably accurate protein structure models, and these methods are far more computationally expensive than ligand-based algorithms with respect to compound throughput.^12^

Ligand-based virtual screening evaluates each compound solely based on its chemical properties, which can provide higher throughput. These approaches are applicable to a broader range of targets, including proteins with unknown structures or assays that measure perturbations to pathways, phenotypes, or cell populations. Ligand shape-matching algorithms can run faster than traditional docking and scale to current synthesize-on-demand chemical libraries.^14^ However, implementations like FastROCS score compounds only based on similarity to individual active reference compounds, ideally in their bound-state conformations, which are not always available. In contrast to supervised machine learning, FastROCS is a shape-based similarity search and does not directly leverage features learned from known inactive compounds.

Other ligand-based virtual screening models are often formulated as a supervised learning problem.^15,16^ Modeling the association between compound structure and activity has a long history^17^ and is referred to traditionally as the quantitative structure–activity relationship (QSAR).^18,19^ Supervised learning formulations of ligand-based virtual screening can be viewed as modern forms of QSAR. Models, such as random forests (RFs), neural networks, and support vector machines,^20−22^ are trained on examples of active and inactive compounds. The requirement for adequate initial assay data is a limitation of the supervised learning approach. However, once trained on sufficient data, these models can evaluate a new compound in milliseconds, making them attractive for very large libraries. Initial prospective evaluations of ligand-based models applied to large libraries have been promising but remain rare.^23−26^ In general, the performance of such models in practical applications with large synthesize-on-demand libraries is still poorly understood. Models that perform well in retrospective evaluations could be inaccurate in synthesize-on-demand libraries where the distribution of chemical structures is different from the training distribution.

Here, we demonstrate that supervised learning approaches commonly used for ligand-based virtual screening are highly scalable and capable of strong prospective performance in very large chemical libraries. We build upon our prior virtual screening effort to find inhibitors of a bacterial protein–protein interaction between PriA and single-stranded DNA binding protein (SSB).^27^ SSB’s interactions are critical for maintaining prokaryotic genome stability and as such represent potential antibiotic targets.^28^ Our previous effort involved the prospective evaluation of an RF model on a library of 22,434 compounds. The RF’s top 250 predictions identified 37 of the 54 active compounds in the library. Here, we examine the applicability and performance of supervised learning models when operating on much larger libraries. Our machine-learning pipeline selects a RF model that successfully prioritizes compounds from over 8 million compounds from Aldrich Market Select (AMS). Then, we successfully apply the model on over one billion compounds in the Enamine REAL database, yielding a small, highly hit-enriched compound subset. These prospective tests demonstrate a cost-effective approach for navigating very large chemical spaces in the search for active compounds. The RF model is easy to train and readily scales to current and future synthesize-on-demand libraries, unlike structure-based approaches.

## 2 Results

Our primary goals were to identify a strong supervised learning model for predicting PriA-SSB inhibitors and then prospectively test the model in a large-scale virtual screen on the AMS and Enamine REAL libraries that contain 8,187,682 and 1,077,562,987 compounds, respectively. As a first step, we used a PriA-SSB high-throughput screening training dataset with 427,300 compounds and 554 actives to compare multiple types of supervised learning algorithms, optimize their hyperparameters, and select the top-performing model. We included classification models (denoted with -C) trained to predict binary activity as well as regression models (denoted with -R) trained to predict continuous % inhibition and identified a random forest classifier (RF-C) as the best model. Compounds in the training dataset were tested at 33.3 μm. Binary activity labels for the classification models were assigned based on an activity threshold of ≥35% inhibition (3 standard deviations in the % inhibition distribution). The training dataset was highly imbalanced, with only 0.13% of the tested compounds meeting the activity criteria.

Next, we assessed the RF-C model’s prospective performance. We compared prioritized compounds from the RF-C model and a similarity baseline model based on chemical structural similarity using the AMS library. After finding that the RF-C model recovered more active compounds and more chemically diverse actives than the baseline, we assessed its scalability to the billion-compound synthesize-on-demand Enamine REAL library. The RF-C model again achieved a high hit rate of 45.6%.

### 2.1 Supervised Learning Hyperparameter Tuning

To determine which supervised learning model would be most effective, we used cross-validation to systematically explore model hyperparameter combinations in five different model classes. The five model classes were RF, eXtreme gradient boosting (XGB), fully connected neural networks (NNs), ensembles, and a similarity baseline. We did not design new supervised learning methods but rather focused on evaluating well-established algorithms. In this stage, we enumerated hyperparameter combinations, training 3080 total models, and then pruned the hyperparameter combinations down to the best 20 per model class (Appendix A.7). We split the training dataset into 10 folds and used only folds 0 through 7 in this stage, reserving the last two folds (folds 8 and 9) for the next stage (Table S1).

**Figure 1:**
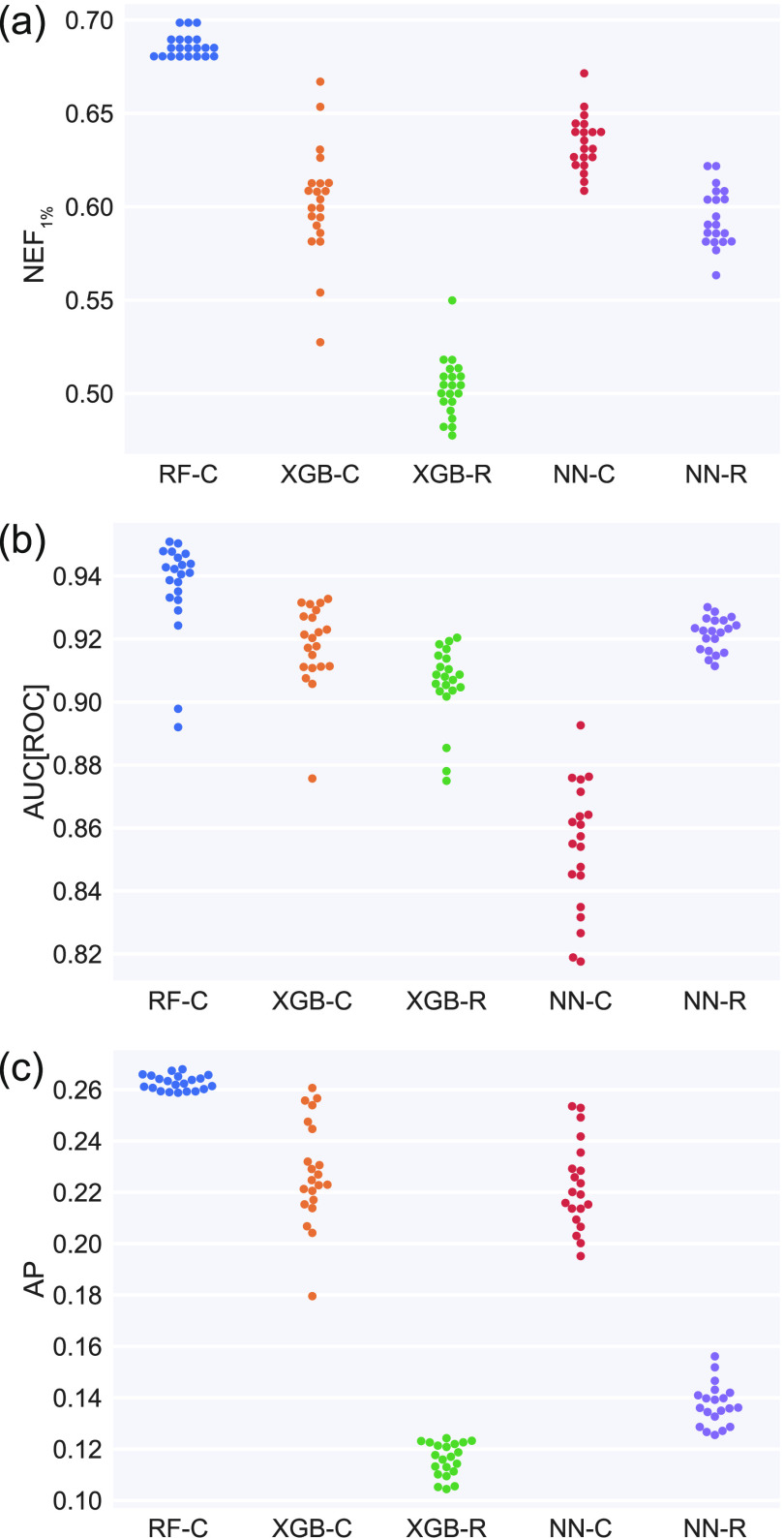
Cross validation mean performance on the top 20 hyperparameter sets from each model class for the (a) NEF_1%_, (b) AUC[ROC], and (c) AP metrics. For the RF-C, XGB-C, and XGB-R model classes, 21 hyperparameter sets are shown due to ties. The -C and -R suffixes denote classification and regression variants, respectively.

Because our focus is on early retrieval of actives, we chose these top models based on mean normalized enrichment factor at 1% (NEF_1%_) performance (Section 4.4). Figure 1 shows the mean performance for the top 20 models (with ties) for each class. The top RF-C models consistently performed better than all other model classes on all three evaluation metrics considered: NEF_1%_, area under the receiver operating characteristic curve (AUC[ROC]), and average precision (AP). The classification versions of the XGB and NN models outperformed their regression-based counterparts. Although it is atypical to model continuous % inhibition instead of binary activity, including both versions of the models enabled us to empirically assess the impact of this modeling choice.

### 2.2 Supervised Learning Model Selection

Next, we compared the performance of the top models from each model class on a reserved test fold. Here, the top 20 (or 21 due to ties) hyperparameter configurations per model class were trained on folds 0–8, with fold 8 used as validation and tested on fold 9. The results for the top model in each class are illustrated in Table 1. In addition to the individual models, we also examined two ensemble methods: model-based ensemble and max vote ensemble. Across classes, the optimal model is an RF-C model that achieved the best performance on two of the three metrics, NEF_1%_ and AP. For prioritizing compounds in a large synthesize-on-demand library, these two metrics are more relevant than AUC[ROC]. NEF_1%_ explicitly focuses on early hit retrieval, which is the goal when selecting a small fraction of compounds from a large library. AUC[ROC] can provide an overly optimistic summary of performance when there are far more inactive than active compounds,^29^ which is the case in high-throughput screening. Therefore, we selected the RF-C model and the similarity baseline for prospective testing. Although the similarity baseline model was outperformed by most of the supervised learning approaches, we included it in the AMS prospective testing as a control. It represents a typical strategy for prioritizing compounds to test from chemical libraries. The similarity baseline simply prioritizes the closest analogues of known actives and is related to standard approaches known as “hit expansion” or “analogue by catalogue.”^4^

**Table 1:** Top Performance across Model Classes on the Test Fold 9^a^

Model	hyperparameter ID	AUC[ROC]	AP	NEF_1%_
RF-C	14	0.89	0.19	0.64
XGB-C	140	0.92	0.17	0.58
XGB-R	81	0.88	0.05	0.42
NN-C	47	0.83	0.13	0.58
NN-R	191	0.90	0.07	0.49
similarity baseline		0.81	0.09	0.40
model-based ensemble	0	0.94	0.17	0.62
max vote ensemble	0	0.94	0.17	0.62

a The best model is an RF-C model in the two most relevant performance metrics AP and NEF_1%_.

To further explore why the ensemble methods did not improve the performance over the best individual models (Figures S1 and S2), we examined the overlap in actives retrieved between the ensembles and top models (Tables S2–S6). Although the ensembles found some actives not prioritized by the best RF-C model, the RF-C found many more actives, leading to better performance. We note several methodological improvements that could improve the ensemble performance in Appendix A.8.

### 2.3 AMS Prospective Screening

Next, we applied the RF-C and similarity baseline models in a prospective test on Sigma-Aldrich’s AMS library, which consisted of 8,187,682 compounds that are mostly in stock. First, the RF-C and similarity baseline models were re-trained using all 10 folds. Then, each model was applied to score the AMS library. The top 1500 ranked compounds from each model were filtered based on cost, delivery, and availability criteria (Section 4.8). The union of these two prioritized and filtered sets comprised 1028 unique compounds, which we purchased from Sigma-Aldrich. The average cost, including dissolution in DMSO and plating, was approximately $40 per compound.

Upon receipt of the plated compounds, we determined that four compounds were incompletely dissolved. These compounds were removed from further consideration, leaving 1024 for testing. Among the 1024 compounds, 701 compounds were in the top 1500 from the RF-C and 705 were in the top 1500 from the similarity baseline, including 382 compounds selected by both models. All 1024 compounds were tested in duplicate at a concentration of 33.3 μm to reveal 412 hits. Our AMS hit criteria required that both replicates exceed 50% inhibition and the compound passed a pan-assay interference compounds (PAINS) filter (Section 4.8).

#### 2.3.1 Prospective Hit Summary

The virtual screening methods had far superior hit rates for the 1024 AMS compounds than would be expected from random selection (Table 2). The training set consisted of 427,300 compounds with 554 actives, a hit rate of only 0.13%. If the hit rate is similar in the AMS library, we would expect only one hit in random selections of 1024 compounds. Both computational models were given similar budgets: 701 and 705 compounds for RF-C and similarity baseline, respectively. RF-C outperformed the similarity baseline, finding 337 hits compared to the baseline’s 256. Both models prioritized a common set of 382 compounds and thus identified the same 181 hits, but the RF-C model recovers more hits (Table 2). Overall, the two models rank the 8,434,707 compounds differently with a Spearman’s rank order correlation coefficient of −0.083.

**Table 2:** Overlap and Hit Rates of the AMS Compounds Selected by the RF-C and Similarity Baseline Models

selector	count	hits	misses	hit rate (%)
RF-C or similarity baseline	1024	412	612	40.23
RF-C	701	337	364	48.07
similarity baseline	705	256	449	36.31
RF-C and similarity baseline	382	181	201	47.38
RF-C but not similarity baseline	319	156	163	48.90
similarity baseline but not RF-C	323	75	248	23.22

**Figure 2:**
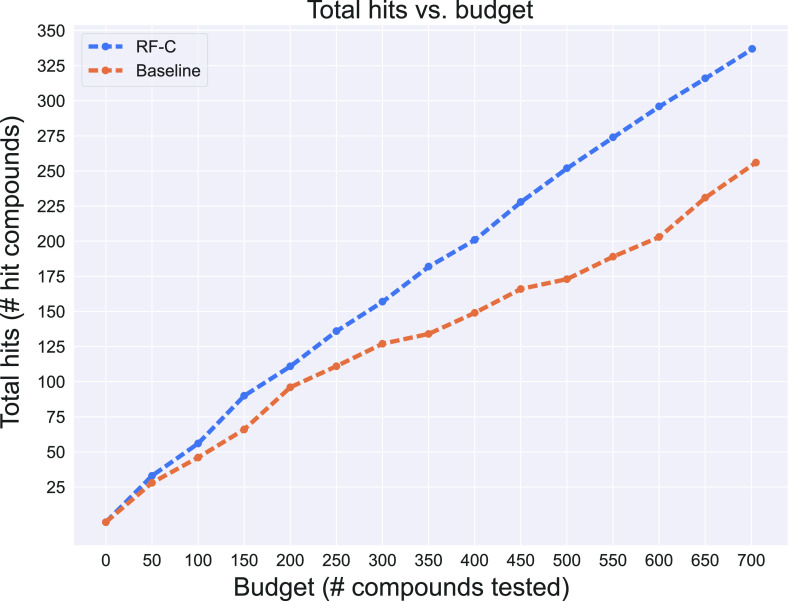
Number of AMS hits identified by the RF-C and similarity baseline models for different compound budgets. The RF-C performance is better than the similarity baseline for all compound budgets, and the gap increases with the budget. The budget is the number of top-ranked compounds evaluated. The total hits are the actives that would be found by screening only those compounds.

Figure 2 shows the hit accumulation as each model is provided a progressively larger hypothetical screening budget. The screening budget is based on the number of compounds selected by each model in descending order of score but not the compound price. The RF-C model consistently outperforms the similarity baseline, and the gap widens as the budget increases. It is expected that this gap would eventually close as the budget expands due to the finite active compounds in the AMS library. However, for practical, cost-effective virtual screening, early hit retrieval performance at small budgets is most relevant.

#### 2.3.2 Chemical Diversity of the AMS Active Compounds

The ideal virtual screening algorithm would not only prioritize many active compounds but also identify chemically diverse or structurally novel hits. Clustering compounds by chemical structure provides one way to assess chemical diversity. We clustered the union of the training set of 427,300 compounds and the 1024 AMS compounds with a custom implementation of the Taylor–Butina^30,31^ method using Tanimoto distances between the compounds’ fingerprint representations, the same chemical features used by the models. The Tanimoto distance is defined as 1 – Tanimoto similarity. We defined two chemical diversity metrics: unique cluster hits and novel cluster hits. We identified the clusters with at least one AMS hit regardless of whether or not these clusters contain training set compounds. This cluster count is defined as the unique cluster hits metric, which gives a measure of the hit diversity. In addition, we calculated the novel cluster hits metric, which is the number of clusters that contain AMS hits but do not contain any training set hits. Novel cluster hits measures how well a model was able to generalize to active chemotypes that were either unexplored or showed no activity in the training dataset. This is similar to an existing novelty measure.^32^Table 3 summarizes these cluster hit metrics at a clustering distance threshold of 0.4 (see Table S7 for other thresholds). The RF-C model substantially outperforms the similarity baseline in both hit diversity metrics, finding twice as many novel chemical clusters.

**Table 3:** Summary of Cluster Hits Metrics on the 1024 AMS-Selected Compounds^a^

selector	unique cluster hits	novel cluster hits
RF-C or similarity baseline	169	72
RF-C	142	61
similarity baseline	115	30
RF-C and similarity baseline	88	19
RF-C but not similarity baseline	72	44
similarity baseline but not RF-C	40	13

a Unique cluster hits denotes the number of clusters containing at least one hit. Novel cluster hits denotes the number of clusters containing AMS hits and no active compounds from the training set. Taylor–Butina clustering with a distance threshold of 0.4 was used to compute clusters.

There was an association between the clusters defined using chemical structures and a chemical’s % inhibition. We categorized compounds as weak, moderate, or strong actives based on % inhibition ranges (Appendix B.2 and Table S8) and counted the number of weak, moderate, and strong actives in each cluster. The different categories of active compounds did not distribute uniformly across the chemical clusters (Fisher’s exact test *p*-value 0.0005). Most strong and moderate actives concentrated in a few clusters.

**Figure 3:**
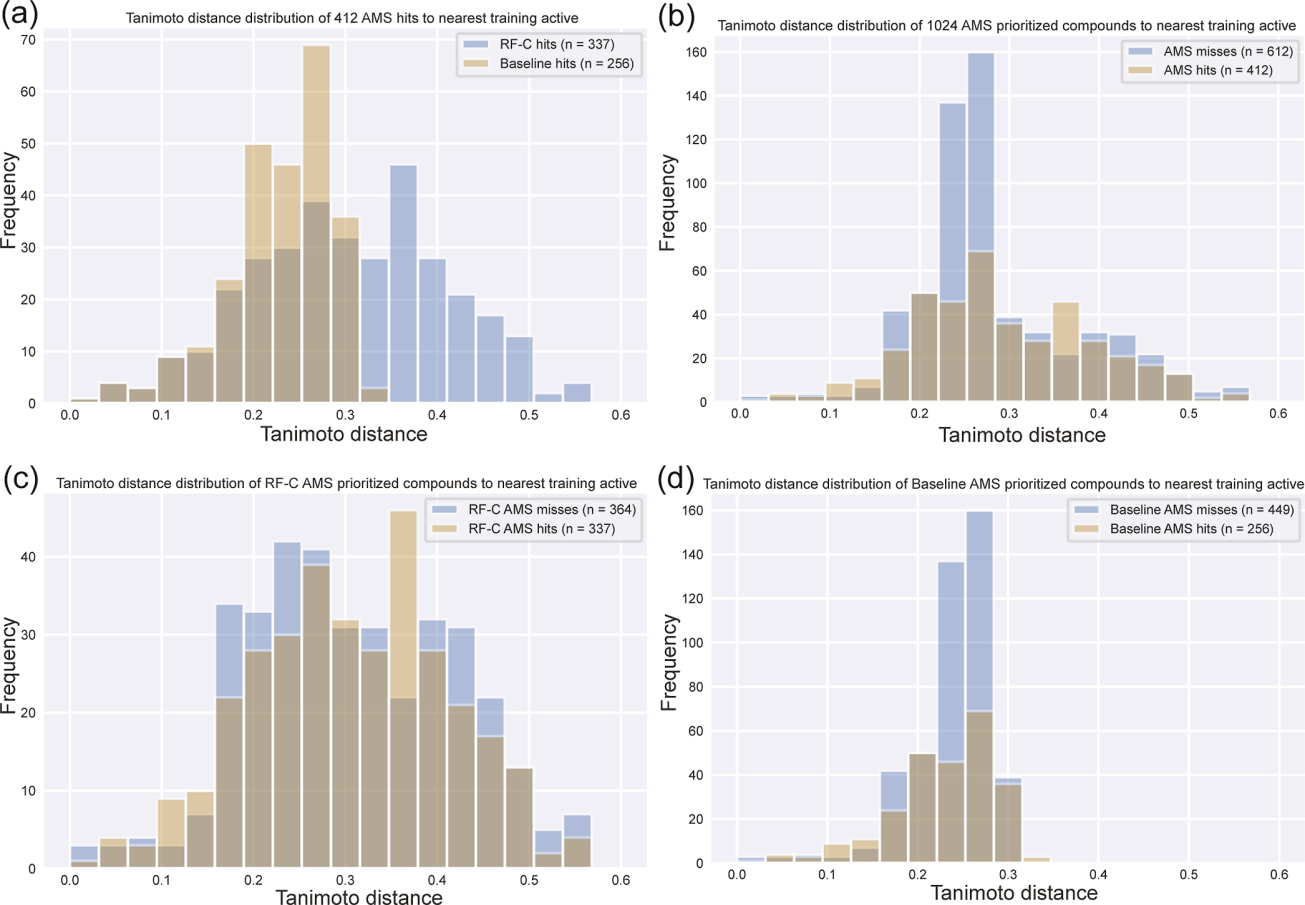
Tanimoto distance distributions of the 1024 AMS compounds to the nearest training set actives. The histograms show distributions of (a) 412 AMS hits based on the model (RF-C or similarity baseline), (b) 1024 AMS compounds based on hits and misses, (c) 701 RF-C AMS compounds based on hits and misses, and (d) 705 similarity baseline AMS compounds based on hits and misses. The Tanimoto distance is 1 – Tanimoto similarity.

In addition to identifying more active compounds than the similarity baseline, the RF-C model also prioritized compounds that are less similar to the training set actives. The Tanimoto distances from the RF-C model’s active prioritized compounds to the most similar training set actives tended to be larger than those from the similarity baseline (Figure 3a). Inspecting the five least similar, experimentally confirmed hits from the RF-C model (Table S9) and the similarity baseline (Table S10) illustrates these differences. The maximum Tanimoto distance from RF-C is 0.57 compared to a maximum distance of 0.32 for the similarity baseline. The similarity baseline is based solely on chemical similarity to the known actives, so its prioritized compounds are mostly minor variations on these actives. In contrast, the RF-C model uses information from both active and inactive training instances to rank compounds. Although there are common substructures with the known actives, the AMS hits from RF-C are more distant overall. The distance between the prioritized AMS compounds and their nearest known active was not predictive of whether the prioritized compound would be active. The distance distributions for AMS actives and inactives were similar when considering all 1024 ordered compounds (Figure 3b), the compounds selected by the RF-C model (Figure 3c), or the compounds selected by the similarity baseline (Figure 3d).

### 2.4 Enamine REAL Prospective Screening

**Figure 4:**
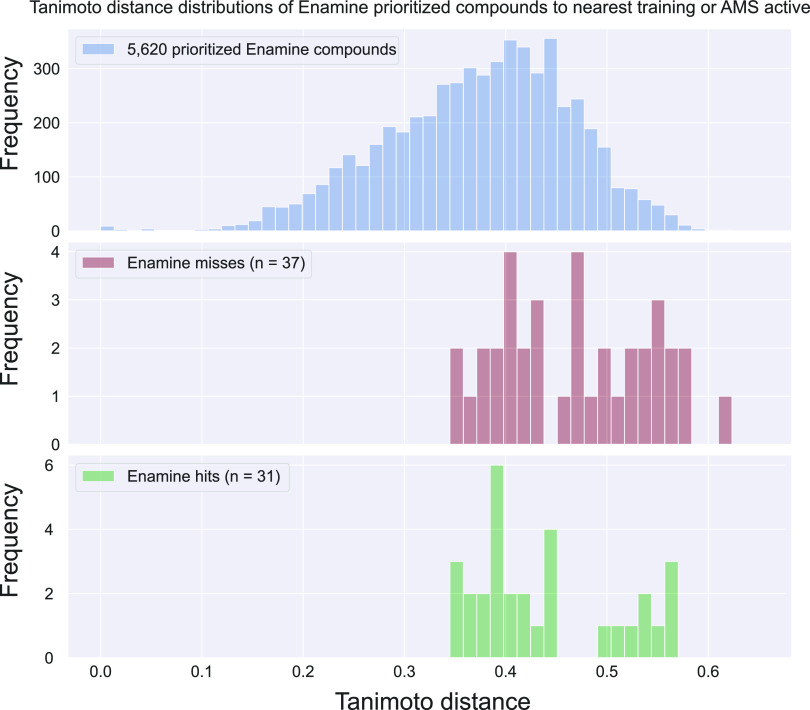
Tanimoto distance distribution of the 5620 prioritized and 68 screened Enamine REAL compounds to their nearest training dataset or AMS active. Middle and bottom panels show the Tanimoto distance distributions of the inactive and active screened compounds using the initial hit criteria. The Tanimoto distance is 1 – Tanimoto similarity.

We used Enamine REAL to demonstrate how our supervised learning workflow can scale to a much larger synthesize-on-demand chemical library, maintain high predictive accuracy, and recover potent active compounds. Scoring all 1,077,562,987 compounds in the library with the RF-C model required only tens of hours when parallelized on 18 CPU cores with modest resources (Appendix B.3 and Table S11). We sorted all compounds by their RF-C score and requested a quote for the top-ranked 10,000 compounds. 5620 of the 10,000 could be delivered in less than a month, and we discarded the rest. Figure 4 (top panel) shows the Tanimoto distance distribution of these 5620 compounds to their nearest training or AMS active. Several Enamine compounds closely resemble known active compounds from the training dataset with distances as low as 0.0. However, many compounds are farther from their closest training set or AMS active with distances exceeding 0.5. Because these compounds already represented the top 0.001% of activity predictions from Enamine REAL, we next optimized the chemical diversity and distance from the known actives (Section 4.9) and selected 68 for screening. These 68 screened compounds had Tanimoto distances to the most similar active ranging from 0.35 to 0.62 (Figure 4).

We also applied the similarity baseline to Enamine REAL and compared the overlap between the RF-C and similarity baseline predictions in order to demonstrate that these same Enamine compounds would not be prioritized by a simpler hit expansion strategy. We examined the overlap between the top *K* RF-C compounds and the top *K* similarity baseline compounds from Enamine REAL for thresholds *K* ranging from 50 to 100,000. The overlaps in prioritized compounds varied from 19 to 34% (Table S12). These overlaps in predicted Enamine compounds are even lower than the 54% overlap the two methods had in the AMS dataset (Table 2), most likely because of the much larger size of the Enamine synthesize-on-demand library.

#### 2.4.1 Enamine REAL Hits

**Figure 5:**
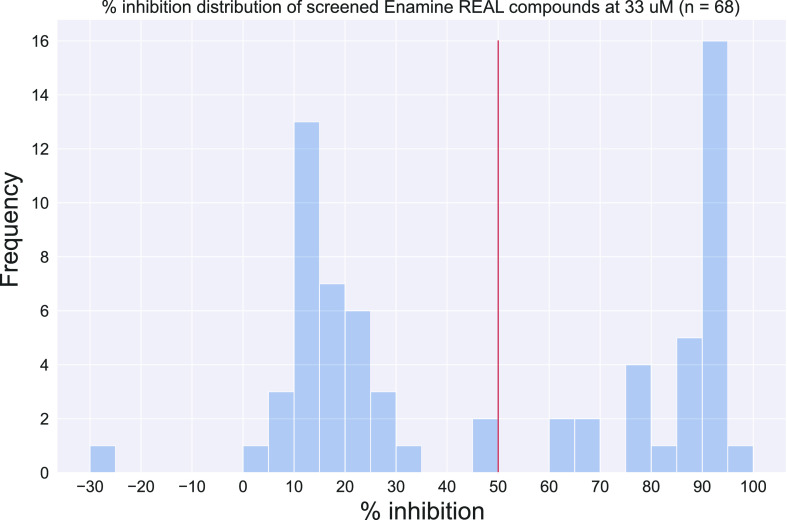
Median % inhibition distribution of the 68 screened Enamine REAL compounds at 33 μm. Compounds with % inhibition of at least 50 (red line) are considered initial hits.

To determine the hits for the 68 Enamine REAL compounds, we generated dose–response curves. We first applied the same hit assignment criteria as the AMS screen and identified 31 initial hits, a 45.6% hit rate (Figure 5). The training set had a 0.13% hit rate, so a random selection of 68 compounds would be expected to yield no hits. We intentionally selected top scoring compounds from different clusters in the prioritized Enamine set, which were also distinct from clusters with active training or AMS compounds, so that the 31 active compounds each represent a unique cluster by design. Similar to the AMS screen, the distributions of the Tanimoto distances to the most similar known active of the Enamine active and inactive compounds are not substantially different (Figure 4).

When we examined the full dose–response curves, we confirmed 28 compounds as hits with IC_50_ values ranging from 1.3 to 37.8 μM (Table 4). Half of these hits had IC_50_ values of 10.0 μM or less. To check for potential false positive hits, we applied computational filters that assess chemical structures that may interfere with the AlphaScreen assay or be histidine mimetics or nickel chelators.^33^ 17 of the 28 hits were flagged with these filters (Table 4). Although the filters suggest caution when interpreting these particular hits, they do not guarantee that the hits are false positives. Structure-based filters are imperfect. Many inactive compounds also matched the filters—6 from Enamine (Table S13), 149 from AMS, and 3281 from the training dataset—even though these could not have interfered with the assay.

**Figure 6:**
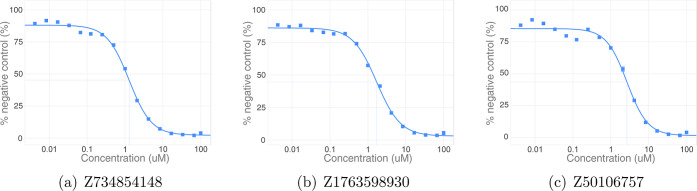
Dose–response curves for the three Enamine compounds with the lowest IC_50_ values.

The three most potent Enamine compounds (Figure 6) share the most similar known active compound, which is from the training set. These three and the nearest active training instance have a common scaffold comprising pyridine and pyrimidine rings spanned by a hydrazone linker. Chemical differences reside on the 4- and 5-positions on the pyrimidine substituent, ortho and para to the linker. Whereas the training compound has 4-chloro and 5-amino substituents, Z1763598930 has only a 5-chloro substituent. In Z734854148 and Z50106757, these positions serve as bridge carbons in purine and thienopyrimidine bicycles, respectively. Several of the confirmed hits contain the 1,10-phenanthroline substructure, which we previously identified as an active compound. However, the new Enamine actives show functional tolerance to substantial additions to this shared substructure with Tanimoto distances to the nearest known active phenanthroline scaffold exceeding 0.5.

Even though the RF-C model was only given a budget of 68 compounds to explore the billion-compound Enamine library, it identified tens of active compounds. These active compounds share common core substructures with their nearest active compounds in the training data and AMS screen. However, they frequently depart significantly with respect to their peripheral substituents (Table 4). Many of the prioritized molecules exhibit well-tolerated, and often substantial, additions to active base scaffolds that might otherwise be missed by simple similarity searches due to larger Tanimoto distances to the nearest active training instances. The RF-C model uses information about both the active and inactive instances in the training data when prioritizing compounds, and an illustrative inactive compound is displayed for each Enamine hit in Table 4. The large dataset sizes and these example inactives suggest that there are likely many other inactive training compounds similar to the Enamine compounds prioritized by the RF-C model, yet the model is nuanced enough to identify 28 confirmed actives among the 68 compounds it prioritized. Although it is imperfect, the RF-C model has some weak capacity to discern active from inactive compounds with similar scaffolds in local chemical space.

Examining the 40 inactives among the 68 Enamine compounds (Table S13) provides some insights into the RF-C model’s errors. Overall, the inactive compounds have approximately similar Tanimoto distances to their nearest active and nearest inactive compounds from the training dataset or AMS compounds as the 28 confirmed active compounds. Therefore, it is unlikely that the model is making erroneous activity predictions for these compounds because their chemical structures and Morgan fingerprint features are from a substantially different distribution than the training distribution. Activity cliffs, which are structurally similar compounds with vastly different activity, are challenging for all supervised learning models and a common source of prediction error.^34,35^ However, these 40 Enamine inactives generally are less similar to the active training instances than compound pairs that would be considered to form activity cliffs.^34^

## 3 Discussion

Despite the many advances in machine-learning approaches for ligand-based virtual screening, few of these algorithms and pipelines have been validated with prospective chemical screens in comparison with appropriate baseline methods.^36^ Machine-learning and virtual-screening tools are now being applied to score and prioritize millions to billions of compounds more routinely.^14,37−39^ There are examples of experimentally evaluating those predictions,^11−13,23−26,40−45^ but they primarily employ structure-based virtual screening workflows. The strong results in our prospective evaluation of PriA-SSB inhibitor predictions support the potential for similar supervised learning-based virtual screening in other drug discovery campaigns. The RF model we selected generalized well to the AMS library of over 8 million compounds and the synthesize-on-demand Enamine REAL library with over a billion compounds. These putative active compounds require further validation, such as testing in our fluorescence polarization assay^28^ as a confirmatory screen. The presence of false positive compounds in the training data, such as AlphaScreen frequent hitters,^33^ may have led to prioritizing similar false positives in the AMS and Enamine REAL collections. 182 out of 412 AMS hits and 17 of the 28 confirmed Enamine hits matched computational AlphaScreen frequent hitter filters,^33^ suggesting that some fraction of our hits would be removed by a confirmatory screen. Nevertheless, the high initial hit rates, potency, and chemical diversity of our prospective screens are quite encouraging. The next steps after validating our AMS and Enamine hits would include screening and optimizing for specificity, toxicity, metabolic stability, and bacterial growth inhibition.

Our PriA-SSB case study revealed some challenges of virtual screening on large commercial chemical libraries and limitations of our current approach. Compound availability in large libraries can change, especially when manually obtaining quotes for the desired compounds. Real-time, automated access to available compounds and prices could improve the screening process. Despite this, high synthesis success rates are an advantage of our strategy of scoring synthesize-on-demand libraries. 68 of the 90 compounds we requested from Enamine were synthesized successfully. In contrast, most generative machine-learning models^46−48^ may propose appealing compounds that are difficult or impossible to synthesize. An additional limitation is that we were unable to directly compare the potencies of the active compounds in the training dataset and the hits found in the AMS and Enamine libraries. We only generated dose–response curves for the Enamine compounds because there were tens of hits from Enamine versus hundreds in the training data and AMS library. If we did confirm the AMS hits with dose–response curves, the hit rate in the prospective AMS evaluation would decrease.

Diversity of hits is important because it provides more potential starting points for hit-to-lead optimization. Our RF was trained only to predict inhibitors and was not concerned with chemical diversity. For the AMS library, we did not filter the selected compounds based on diversity. Rather, we ranked them by predicted activity score and assessed chemical diversity post hoc. When moving to larger synthesize-on-demand libraries like Enamine REAL, diversity requires more formal attention. There are many highly similar or redundant compounds, so prioritizing based on predicted activity alone can concentrate the selected compounds in an undesirably small number of large chemical clusters. When selecting Enamine REAL compounds, we used heuristics such as cluster membership and Tanimoto distance from known actives to promote diversity. Related work prioritized the compound with the highest predicted score per cluster.^25^

We primarily used Taylor–Butina clustering^30,31^ to quantify chemical diversity because it groups compounds with similar chemical structures, but there is no universally accepted way to assess the structural diversity within a compound set. Taylor–Butina clustering, which readily scales to large compound sets, is susceptible to the order in which the compounds are processed and can result in different clusterings. For example, an active may have been clustered earlier than an inactive that is within the cutoff distance. To avoid potential dependence of our diversity assessment on the choice of distance threshold used in Taylor–Butina clustering, we applied three different thresholds and observed consistent diversity differences between compound sets. The RF-C model outperformed the similarity baseline in both unique and novel cluster hits (Tables 3 and S7), confirming that it can prioritize novel chemical structures that are not present in the training data. Even though all of the compounds we ordered from Enamine belonged to different chemical clusters, some did contain the same core scaffold (Table 4).

**Table 4:**
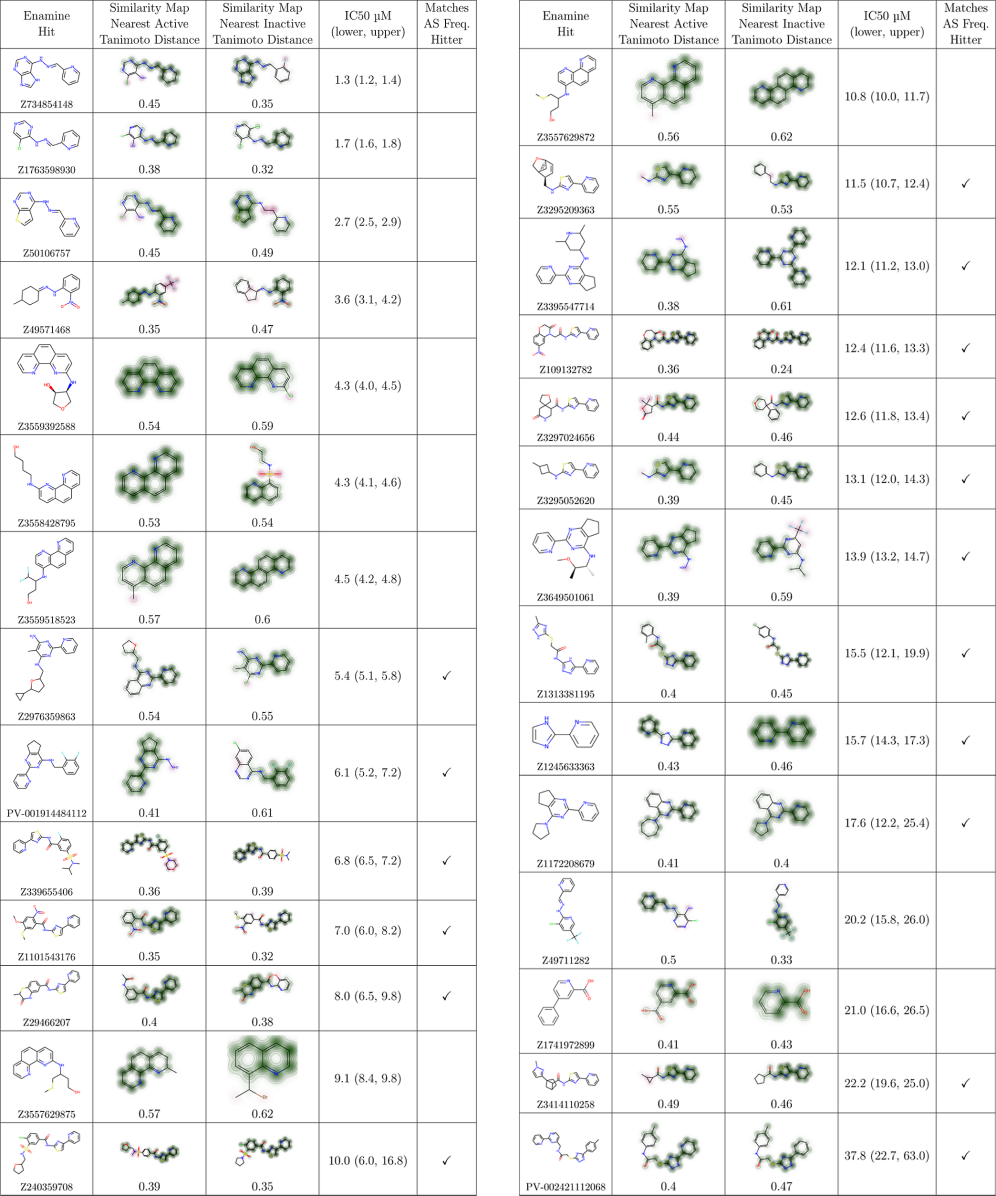
28 Confirmed Hits from the Enamine REAL Compounds Based on Dose–Response Curves^a^

a The most similar known active compound from the training set or AMS compounds is shown along with its Tanimoto distance (which is 1 – Tanimoto similarity) to the Enamine hit. The similarity maps from RDKit show similar substructures in green and dissimilar substructures in red. The IC_50_ values are shown along with the 95% lower and upper confidence limits. Hits that match AlphaScreen frequent hitters filters^33^ are denoted with a checkmark.

There are open questions about when our approach to virtual screening on large compound libraries is applicable. In this study, we had access to a large training set with 427,300 screened compounds and 554 actives. A typical target will have far fewer screened compounds and known actives at the start of the drug discovery process. Virtual screening will have greater impact if it can succeed without requiring an initial high-throughput chemical screen to train models. Having established that our supervised learning virtual screening was successful with an ideal training set, future work can explore the tradeoff between training set size and prospective accuracy in compounds selected from synthesize-on-demand libraries. We were unable to explore that tradeoff in this study because executing multiple rounds of prospective testing for different training set sizes would greatly inflate the budget required to order compounds and test predictions. However, related work that prospectively evaluated supervised learning for virtual screening provides optimism that similar approaches can succeed with much less training data. The DNA-encoded libraries in McCloskey et al.^25^ used training sets with similar sizes to ours, but other studies trained on fewer examples: 1186,^26^ 2335,^24^ 7684,^49^ or 45,127^23^ compounds. The most important difference between our work and these studies is that ours applies supervised learning to the largest prospective chemical library and directly assesses the challenges of prioritizing compounds in synthesize-on-demand libraries.

Our study considered only a limited set of machine-learning models, hyperparameters, and chemical representations. These choices were partially influenced by our prior experience modeling PriA-SSB inhibitors.^27^ Sheridan et al. previously identified XGB hyperparameters that performed well across a variety of QSAR tasks,^50^ which could have guided our XGB hyperparameter selection. In addition, our range of values for the XGB hyperparameter that controls class weights was a poor fit for our highly imbalanced training dataset, which may have contributed to XGB underperforming RF. More notably, we did not include any graph neural networks. Graph neural networks, ranging from convolutional neural networks on the molecular graph^51,52^ to more advanced graph transformers^53^ are appealing because they do not require precomputing chemical representations such as Morgan fingerprints. Expanding the set of machine-learning models, hyperparameters, and chemical representations evaluated could have affected our retrospective cross-validation results and led to the selection of a different model. However, our primary goal was not model benchmarking but rather studying the prospective performance of the best model selected by cross-validation in large commercial libraries. Future studies that include more machine-learning models during cross-validation may select even stronger models that provide better prospective performance than we observed.

Accurate virtual screening algorithms are essential for expanding into synthesize-on-demand chemical libraries in which purchasing and screening all compounds is impossible. Synthesize-on-demand libraries can provide access to many more hits and higher quality hits with respect to chemical diversity and other desirable properties, as exemplified by the potent PriA-SSB inhibitors we identify. Our simple supervised learning approach has a high hit rate, good chemical diversity, and can scale to synthesize-on-demand libraries. Scoring over one billion compounds takes less than 1000 CPU hours and can be trivially parallelized to score even larger chemical libraries in the future. In contrast, the computational costs of structure-based scoring of a library of this magnitude are significant if not prohibitive. One recent structure-based study required approximately 5 million CPU hours to evaluate 1.3 billion compounds.^12^ Another used 27,612 GPUs to score 1.37 billion compounds in less than 24 h.^39^ However, active learning^54^ and fragment-based docking strategies^13,44^ are emerging to avoid exhaustively docking entire synthesize-on-demand libraries.

Virtual screening of ultra-large synthesize-on-demand libraries presents both opportunities and new challenges.^55^ One concern is the possibility of getting buried by false positives due to biases or errors in the scoring model that go undetected during model validation.^56,57^ Models may erroneously recognize uncommon or exotic chemical features as important to activity due to limitations or mislabeled instances in a ligand-based model’s training set or unsuitable parameters in a scoring function. Compounds with problematic features could be rare enough in smaller libraries that they account for only a small number of false positives and go unnoticed during model development. However, the sheer size and chemical diversity of synthesize-on-demand libraries could cause the top scoring compounds to be dominated by such errors.

We are encouraged that these potential issues did not arise in our large-scale ligand-based virtual screen (Tables 4 and S13) or the largest structure-based virtual screens noted above. Although innovative algorithms will continue to advance virtual screening capabilities, probing the practical challenges and potential benefits of computationally guided screening in synthesize-on-demand chemical libraries necessitates prospective experimental testing.^58^ The success of our two prospective screens showcases supervised learning as a powerful approach for navigating large synthesize-on-demand chemical libraries in future drug discovery campaigns.

## 4 Methods

### 4.1 PriA-SSB Training Datasets and Chemical Screening

We trained virtual screening models on new and previously generated datasets for the PriA-SSB target.^27,28^ This target is a protein–protein interaction that is involved in bacterial DNA replication restart^59^ and was considered as a candidate target for antibiotics development.^28^ The compounds in these datasets come from four batches of a Life Chemicals Inc. (LC) library designated LC1–LC4 and the Molecular Libraries Probe Production Centers Network (MLPCN) library (Table 5). The compounds considered for prospective evaluation come from the in-stock AMS library and the synthesize-on-demand Enamine REAL library.^60^

**Table 5:** Chemical Libraries and Compound Counts^a^

stages	library	# compounds
pre-processing	LC123 (primary and retest)	74,763
pre-processing	LC4 (primary)	25,278
pre-processing	MLPCN (primary and retest)	337,104
hyperparameter tuning, model selection, prospective	training set	427,300
prospective	AMS	8,187,682
prospective	Enamine REAL	1,077,562,987

a The training dataset is merged from the LC1234 and MLPCN libraries.

We screened the MLPCN compound library using an AlphaScreen (AS) assay at a single concentration (33.3 μm) following the same protocol previously used to screen the LC1234 libraries.^27,28^ Briefly, library compounds and controls were dispensed from 10 mM DMSO stocks into white 1536-well plates with an Echo 550 acoustic liquid handler. Next, 3 μL of a master mix was added to each well using a Mantis liquid handler with a high-volume silicone chip to yield a final reaction mixture containing 10 mM HEPES-HCl (pH 7.5), 150 mM NaCl, 1 mM MgCl_2_, 10 mM dithiothreitol, 1 mg/mL bovine serum albumin, 0.01% Triton X-100, 0.1 μM *Klebsiella pneumonia* PriA (with N-terminal 6×His tag), 0.1 μM Biotinylated-SSBct (N-Biotin-Trp-Met-Asp-Phe-Asp-Asp-Asp-Ile-Pro-Phe-C), 5 μg/mL of both AS acceptor and donor beads, and 33.3 μm of compound. For positive and negative control wells, the compound was replaced with 25 μM SSBct peptide (N-Trp-Met-Asp-Phe-Asp-Asp-Asp-Ile-Pro-Phe-C) or 25 μM ΔFSSBct (N-Trp-Met-Asp-Phe-Asp-Asp-Asp-Ile-Pro-C), respectively. Plates were centrifuged briefly and rocked for an hour at room temperature. Then, the AS signal of each well was measured with a PheraStar plate reader using a 0.1 s settling time, a 0.3 s excitation, a 0.04 s delay, and a 0.6 s integration time. AS signals for each well were adjusted as previously described to reduce the impact of plate and edge effects.^28^ We calculated a *Z*′ factor for each plate^61^ and repeated plates with *Z*′ < 0.5. In addition, we calculated the % inhibition relative to the controls for each compound. We retested compounds with ≥35% inhibition that also passed PAINS filters^62,63^ with the same assay to confirm activity.

We compiled the training dataset by merging the MLPCN screening data with the LC1234 screening data (Table 5). We defined active compounds, also referred to as hits, as those with median % inhibition of the primary screens ≥35%, median % inhibition in retest screens ≥35%, and not flagged by the PAINS filter (Appendix A.1). The 35% single point activity threshold used for active label assignment corresponds to 3 standard deviations in the % inhibition distribution observed in the LC1234 and MLPCN screens. The merged training dataset contained 427,300 compounds with 554 actives. We split the training data into 10 folds for cross-validation. The splitting method takes into account library information (LC1234 and MLPCN) by grouping the compounds based on their libraries and then stratifying each of these groups into 10 folds that maintain roughly the same binary activity label distribution. The splitting does not account for shared chemical structures across folds. Chemical structural similarity across training and testing folds can inflate performance in retrospective benchmarking, and creating folds based on chemical clusters or scaffolds can partially, but not fully, alleviate that bias.^64^ However, in our study, we do not emphasize the cross-validation performance metrics and instead use the prospective evaluations to assess hit rates and generalization.

### 4.2 Chemical Feature Generation and Clustering

We used RDKit Morgan fingerprint features^65,66^ for all virtual screening methods. We converted each compound SMILES to an RDKit mol object, removed salt counter ions using RDKit’s SaltRemover function, and generated a Morgan fingerprint with length 1024 and radius 2. Using the fingerprints, we then defined chemical relationships based on the Tanimoto distance, which is equivalent to Jaccard distance and also defined as 1 – Tanimoto similarity. To judge chemical diversity, we used Taylor–Butina^30,31^ clustering at various Tanimoto distance thresholds (0.2, 0.3, and 0.4). Taylor–Butina takes a distance threshold as the input that forces compounds within the same cluster to be within that threshold. Table S14 summarizes the number of compounds, number of clusters, and number of unique clusters that are in each cross-validation fold. The number of unique clusters that are in a given fold but not the others can measure a virtual screening model’s ability to generalize. Because we generated cross-validation folds by splitting on library and activity instead of cluster membership, the clusters are not uniformly distributed across folds.

### 4.3 Virtual Screening Methods

We considered a variety of supervised learning ligand-based virtual screening algorithms. Most algorithms could be trained in a classification setting to predict binary activity as well as a regression setting to predict the % inhibition, which is indicated by appending -C or -R, respectively, to the model abbreviation.

#### 4.3.1 Random Forest

An RF model consists of a collection of base learners, typically decision trees.^21^ Each base learner is trained on random subsamples of the training data with replacement. This process is known as bootstrap aggregation (bagging), which helps to combat overfitting.^67^ Furthermore, individual decision trees are split on random subsets of the features, which further helps to combat overfitting.^21,68^ Classification is done by aggregating the votes from the base learners. We used the scikit-learn RF implementation.^69^ The RF hyperparameters include the number of base learners, number of features, leaf node samples, and class weights (Table S15). Because RF-R regression models grew too large under some hyperparameter settings, we only considered RF-C classification models.

#### 4.3.2 eXtreme Gradient Boosting

XGB is an ensembling method that is based on the concept of gradient boosting.^70−72^ It builds the base learners sequentially, where each learner is built to reduce a loss function whose terms include the gradients (residuals) of previously built learners. As a result, each consecutively built learner aims to correct the mistakes of past learners using the gradients. We use the XGBoost Python package implementation.^72^ The XGB hyperparameters involve varying the maximum depth, learning rate, and number of estimators (Tables S16 and S17).

#### 4.3.3 Neural Network

An NN consists of a series of hidden layers corresponding to weight matrices followed by non-linear activation functions. The process of forward propagating the input along the hidden layers via matrix multiplication is repeated until a final output layer is reached, which makes a prediction. The weight matrices are trained by gradient descent on the loss of the output; this technique is called backward propagation. Our NN architecture is a fully connected NN, also known as a multilayer perceptron, that takes the Morgan fingerprint bits as input and does not use the molecular graph structure. Our implementation uses Keras^73^ with the Theano backend^74^ and the Adam optimizer.^75^ The NN hyperparameters involve varying the learning rate, drop out, number of hidden layers, number of hidden units, and activation functions (Tables S18 and S19).

#### 4.3.4 Ensembles

The ensemble models combine predictions from multiple existing models, which are referred to as base models. Max vote ensemble applies the max function and outputs the largest value from the base models. The model-based ensemble is a stacking ensemble method ,^76^ which consists of two layers: several base models as the first layer and another classifier as the second layer. In the first layer, multiple classification and regression models (the base models) output the predicted values for each molecule. In the second layer, another classifier (the ensemble model) is trained to balance the output values from the base models. In this way, the simple classifier from the second layer can ensemble the performance from different base models. Finally, all the base models are retrained on the complete training data, passing through an ensemble model to get the final prediction value. Both ensemble methods incorporate a subset of the best models from the hyperparameter tuning stage and have 13 hyperparameter sets that vary which base models are used (Table S20). Details of the ensemble training are described in Appendix A.8 and Figure S3.

#### 4.3.5 Similarity Baseline

The baseline model represents a simple strategy for prioritizing compounds based on their similarity to known actives.^77,78^ It ignores the inactive compounds in the training data. Given the known actives in the training data and a query molecule, the similarity baseline calculates the Tanimoto similarity between each active and the query. The maximum Tanimoto similarity is returned as the query molecule’s score. Therefore, query molecules whose fingerprints are most similar to those of known actives are prioritized.

### 4.4 Performance Metrics

The computational models generate different types of scores, such as the probability that a compound is active or the predicted % inhibition. In all cases, compounds can be sorted by these scores so that those most likely to be active are first. We consider performance metrics that are based only on these compound ranks. For instance, one can move from most likely to be active to least likely, applying thresholds to compute the true positive rate (TPR), false positive rate (FPR), recall, and precision.
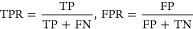
1
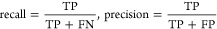
2

Plotting the TPR versus FPR and computing the area under the curve generates the AUC[ROC] metric. The AUC[ROC] serves as a general metric for comparing performance among models as it does not focus on early or late retrieval exclusively. However, as discussed in Section 2.2, AUC[ROC] can be misleading for imbalanced datasets. Similarly, the AP summarizes a precision–recall curve across different thresholds. Using scikit-learn’s^69^ implementation, the AP metric computes the precision *P* and recall *R* at each threshold *k* and then sums as follows
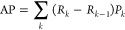
3

AP avoids interpolation issues with computing the AUC of the precision–recall curve.

Enrichment factor (EF) computes the ratio between the number of actives found by a model in its top selected compounds versus a random selection of that many compounds. We normalize EF by the maximum EF possible for a perfect model. Given a fraction *F* ∈ [0, 100%], we compute the normalized enrichment factor at *F* (NEF_*F*_) as follows
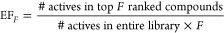
4
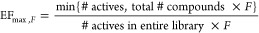
5
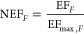
6

We use NEF_1%_ to assess the early retrieval performance in the top 1% of compounds ranked by a model.

### 4.5 Pipeline Overview

We follow a four-stage pipeline: hyperparameter tuning, model selection, and two prospective screening stages. The hyperparameter tuning stage considers many hyperparameter combinations and filters them to the top 20 per model class (with ties). The model selection stage includes the best models from the hyperparameter tuning stage and introduces ensembles that combine these best models. This stage selects a single model for prospective evaluation. The AMS prospective stage uses the best selected model from the model selection stage and the similarity baseline to prioritize compounds from the AMS dataset. The Enamine REAL prospective stage uses that same best selected model from the model selection stage to prioritize compounds from the Enamine REAL dataset. Table 6 summarizes the hyperparameters per stage for each model class.

**Table 6:** Number of Hyperparameter Configurations Evaluated for Each Model Class at Each Stage^a^

model	hyperparameter tuning	model selection	AMS prospective	Enamine prospective
RF-C	216	21	1	1
XGB-C	1000	21		
XGB-R	1000	21		
NN-C	432	20		
NN-R	432	20		
similarity baseline		1	1	
model-based ensemble		13		
max vote ensemble		13		
total	3080	130	2	1

a The pipeline culminates with a single top performing model. The similarity baseline was added as a control in the model selection and AMS prospective stages.

### 4.6 Hyperparameter Tuning

The purpose of the hyperparameter tuning stage is to prune the large number of hyperparameter settings to the top 20 (with ties) for each of the base model classes: RF-C, XGB-C, XGB-R, NN-C, and NN-R. For this stage, we only use the first eight folds of the 10-fold training set. The last two folds are reserved for building ensemble models and assessing test set performance in the subsequent model selection stage. For each hyperparameter setting, we conducted four cross-validation runs with different combinations of the first 8 folds (Table S1). For each cross-validation run, we record the test fold performance on AUC[ROC], AP, and NEF_1%_.

### 4.7 Model Selection

We consider the top 20 (with ties) hyperparameter sets from the hyperparameter tuning stage for each base model class based on mean performance of NEF_1%_. This gives a total of 103 selected models (the 3 additional models are included due to ties). We train all of the candidate models (103 base models, 1 similarity baseline, and 26 ensembles) a single time. We use folds 0 through 7 for training, fold 8 for validation, and fold 9 for testing. This yields a total of 130 performance measures on AUC[ROC], AP, and NEF_1%_. The single top performing model is selected for the two prospective screening stages. In the RF-C model class, three models were tied in NEF_1%_. To break the tie, we used AP to select the best RF-C model.

Our original intention was to select the final prospective model based on fold 9. From Table 1, this would be the RF-C model with hyperparameter ID 14. However, the final prospective model was inadvertently selected based on hyperparameter tuning stage performance instead. This best model was also an RF-C model with hyperparameter ID 139 and fold 9 performance AUC[ROC] = 0.94, AP = 0.17, and NEF_1%_ = 0.62. This RF-C model is still better than the top performers from other model classes on the NEF_1%_ metric that we used to select the model. Furthermore, selecting on the hyperparameter tuning stage performance is still a valid approach to select a model for the prospective evaluation because it uses cross-validation.

### 4.8 AMS Compound Selection and Screening

The AMS prospective stage uses two models: the best performing model from the model selection stage (an RF-C model) and the similarity baseline. Both models are retrained on all 10 folds and then used to predict scores on the AMS library, which contains 8,187,682 compounds after removing compounds that are also in the training data. First, the RF-C and similarity baseline models each select the top 1500 ranked compounds based on their prediction scores. This produces two lists with many compounds present in both lists. In an effort to maximize the number of compounds that could be purchased, we removed compounds that cost > $75 for the smallest sample, had delivery time > 21 days, or came from vendors providing fewer than six compounds in our list. After filtering, we took the union of the remaining top 600 compounds from the RF-C model and the top 600 from the similarity baseline model. We ordered the 1028 unique compounds in the union that were still available for purchase from AMS. When evaluating the hit rate for the RF-C and similarity baseline models, we considered not only their top 600 ranked predictions after filtering but rather all compounds we ordered that were in their original top 1500 ranked compounds. For example, the compound ranked 73 by the RF-C and 986 by the similarity baseline is considered to be predicted by both models in Table 2.

After receiving the 1028 compounds, we conducted two replicate screens following the same protocol described above for the MLPCN library. We excluded four compounds because they could not be dissolved and plated. We analyzed the % inhibition distributions for the 1024 screened compounds for each replicate individually to determine the hit criteria (Figure S4). For a large random screen, we could set the % inhibition cutoff at some standard deviations above the mean.^61^ Thus, we could use the same MLPCN threshold of 35% inhibition. However, these AMS screens were targeted toward likely active compounds. Therefore, we applied a more stringent cutoff of 50%, which is the smaller % inhibition distribution mean from the two replicates (Figure S4). In addition to requiring at least 50% inhibition in both replicates, we also required active compounds to not match a PAINS filter.^62,63^

### 4.9 Enamine REAL Computational Scalability, Compound Selection, and Screening

The Enamine REAL database^60^ contained 1,077,562,987 compounds when we downloaded it on October 11, 2019. To estimate the computational scalability of scoring compounds with the RF-C model from the model selection stage, we split the REAL dataset into 18 batches of about 60.3 million compounds each. Each batch was run as a job on an independent CPU compute node at the University of Wisconsin–Madison’s Center for High-Throughput Computing using HTCondor.^79,80^ Each job processes the batch by generating the chemical fingerprint features and then making activity predictions using the RF-C model. The model was trained only on the training set and not the 1024 AMS compounds.

After scoring the entirety of Enamine REAL, we requested a quote for the top 10,000 ranked compounds. We then pruned the list to 5620 compounds based on availability of starting materials and delivery constraints. We clustered the training set compounds, the 1024 AMS compounds, and the 5620 Enamine REAL compounds using Taylor–Butina with a 0.4 distance cutoff. We used these clusters to select a final list for purchase, seeking compounds that were predicted to be highly active, were chemically diverse, and were chemically dissimilar from known active compounds in the training set and AMS set.

To emphasize novel chemical structures, we retained only Enamine REAL clusters that did not contain an active compound from the training set or the AMS set. We were interested only in clusters that the model predicted to have actives despite not belonging to a cluster with known actives. This filter reduced the Enamine REAL list from 5620 to 2679 compounds.

Next, we retained only compounds that passed the PAINS^62^ filter, Inpharmatica filter, and Lipinski’s rule of five. The PAINS filter used RDKit’s FilterCatalog. The Inpharmatica and Lipinski filters used rd_filters.^81^ This filtering step reduced the Enamine REAL list from 2679 to 1604 compounds. Because over 1000 compounds remained, we further emphasized chemical diversity by only retaining compounds that had a Tanimoto distance ≥ 0.35 from the closest training set active and AMS active. This distance filter reduced the Enamine REAL list from 1604 to 1354 compounds.

For the remaining clusters, we selected the highest scoring predicted active as the representative. This selection reduced the Enamine REAL list from 1354 to 311 compounds. Finally, we selected 100 diverse compounds from the remaining list via iterative Tanimoto distance selection. In the first iteration, the compound with highest activity prediction was selected. Then, the greedy method iteratively selected the candidate compound that was most distant to the already selected compounds until 100 compounds were selected.

We requested an updated quote for these 100 compounds. Only 90 of the 100 selected compounds were still available in stock or in the REAL database. The other 10 required custom chemistry, which is more expensive and has a longer delivery time. We ordered the 90 that did not require custom chemistry. Synthesis failed for 22 of the 90 compounds, so we screened 68 Enamine compounds against PriA-SSB.

All 68 compounds were initially screened in four replicates at eight concentrations ranging from 515.6 nm to 66 μm. We defined compounds whose median % inhibition at 33 μm was at least 50%, the same threshold used for the AMS screen, as initial hits. We repeated the dose–response curve screens for two additional rounds of ten compounds each, expanding the range of concentrations tested to improve the quality of the curve fits (Appendix A.9). We defined confirmed hits as those with a dose–response curve in curve class^82^ 1.2 or in curve class 2.2 with an IC_50_ 95% upper confidence limit within the tested range of concentrations. We used the Collaborative Drug Discovery Vault software^83^ to fit dose–response curves, calculate IC_50_ values and the 95% upper and lower confidence limits, and define curve classes.

### 4.10 Code and Data Availability

Our Python implementation and conda environments with the required Python packages are available on GitHub (https://github.com/gitter-lab/pria-ams-enamine) and archived on Zenodo (https://doi.org/10.5281/zenodo.5551235). The chemical screening datasets are available at PubChem (AID: 1272365) and Zenodo (https://doi.org/10.5281/zenodo.5348290).

## Supporting Information Available

Additional details about the dataset preparation, model hyperparameters, ensemble models, compound clustering, Enamine REAL dose–response curves, Enamine REAL prediction timing, and model predictions (PDF)
