# Supplementary material for: Evaluating Scalable Supervised Learning for Synthesize-on-Demand Chemical Libraries
Source: J Chem Inf Model. 2023 Aug 25;63(17):5513–28. doi: 10.1021/acs.jcim.3c00912 (PMC10538940; doi:10.1021/acs.jcim.3c00912)
Supplement: Supplementary file 1 — ci3c00912_si_001.pdf [file ci3c00912_si_001.pdf]

Supporting Information: Evaluating scalable supervised learning for synthesize-on-demand chemical libraries

Moayad Alnammi,[†,‡,¶] Shengchao Liu,[†,‡,@] Spencer S. Ericksen,[§] Gene E. Ananiev,[§] Andrew F. Voter,[‖] Song Guo,[§] James L. Keck,[‖] F. Michael Hoffmann,[§,⊥] Scott A. Wildman,[§] Anthony Gitter[*,†,‡,#]

† Department of Computer Sciences, University of Wisconsin-Madison, Madison, WI 53706, United States

‡ Morgridge Institute for Research, Madison, WI 53715, United States

¶ Department of Information and Computer Science, King Fahd University of Petroleum & Minerals, Dhahran 31261, Saudi Arabia

§ Small Molecule Screening Facility, University of Wisconsin-Madison, Madison, WI 53792, United States

‖ Department of Biomolecular Chemistry, University of Wisconsin-Madison, Madison, WI 53706, United States

⊥ McArdle Laboratory for Cancer Research, University of Wisconsin-Madison, Madison, WI 53705, United States

Department of Biostatistics and Medical Informatics, University of Wisconsin-Madison, Madison, WI 53792, United States

@ Current address: Mila - Quebec AI Institute, Montreal, Quebec H2S 3H1, Canada

* Email: gitter@biostat.wisc.edu

A Supplementary Methods

A.1 Training Data Preprocessing Overview

Here we describe in detail how we preprocessed the LC and MLPCN screening data in Table 5 to create the 427,300 compound training dataset. At the time this dataset was assembled, the LC4 retest dataset was not available so hits were defined using only the LC4 primary screen. The first step involved merging the three primary and two retest screens.

1. **Primary** screen for 74,896 LC123 compounds.

2. **Retest** screen for 2,698 LC123 compounds.

3. **Primary** screen for 25,290 LC4 compounds.

4. **Primary** screen for 337,990 MLPCN compounds.

5. **Retest** screen for 1,400 MLPCN compounds.

Merging these datasets yielded a total of 442,274 entries; a compound could have multiple readings. This dataset was then processed to determine the binary activity of each compound. Specifically, for each compound, the scores were grouped, and three criteria were assessed: the median % inhibition of primary screens was $\geq$ 35%, the median % inhibition of retest screens was $\geq$ 35%, and the compound did not match a PAINS filter.[62,63] A compound was deemed active if it met all three conditions. Finally, the training dataset was constructed such that it contained one row per compound that included its binary activity label and its median inhibition score based on all recorded primary inhibition scores.

A.2 Merged Dataset Structure

Each of the original screening data files had eight columns: **SMSSF ID**, **CDD SMILES**, **Batch Name**, **Library ID**, **Plate Name**, **Plate Well**, **Run Date**, **PriA-SSB AS % inhibition**. We created a combined dataset with the following columns:

1. **Molecule ID**: uniquely identifies a molecule. A non-negative integer.

2. **Duplicate ID**: denotes the duplicate number (replicate). This is done so that the same molecule can have multiple % inhibition readings; allows grouping by 'Molecule ID'.

3. **SMSSF ID**: molecule ID used by the screening facility.

4. **Batch Name**: used at the screening facility for screening batch identification. Not used in this project.

5. **Library ID**: the libraries that contain the molecule: LC1, LC2, LC3, LC4, and MLPCN. Note that a molecule can be in multiple libraries.

6. **Plate Name**: specifies the plate identifier for this molecule. Can help identify retests if **Plate Name** contains the text **CP**.

7. **Plate Well**: specifies the well row-column location of the molecule on the plate.

8. **Run Date**: specifies the date the data was entered into Collaborative Drug Discovery (CDD) software.

9. **CDD SMILES**: the CDD software SMILES string of the molecule.

10. **RDKit SMILES**: RDKit canonical smiles of the molecule. This is used for uniqueness identification of molecules.

11. **MorganFP**: RDKit Morgan fingerprint of the molecule.

12. **PriA-SSB AS % inhibition**: the AlphaScreen-based % inhibition score of the molecule.

13. **PriA-SSB AS Activity**: the binary activity $\{0, 1\}$ of the molecule.

14. **Primary Filter**: is active (denoted as 1) if the median of the primary screens for this molecule is greater than or equal to **binary_threshold**.

15. **Retest Filter**: is active (denoted as 1) if the median of the retest screens for this molecule is greater than or equal to **binary_threshold**.

16. **PAINS Filter**: is active (denoted as 1) if the molecule is not reported as a PAINS compound by the RDKit PAINS filter.

A.3 Ensuring Molecule Uniqueness

There are two types of repeated molecules for which we retain all measurements. The first is molecules that were experimentally tested more than once, which we generally refer to as replicates. Molecules can be tested more than once because they belong to multiple libraries (e.g., LC1 and MLPCN) or they were included in a primary and a retest screen. The other type of repeated molecules has different **SMSSF ID**s but the same **RDKit SMILES** due to salts. These molecules with the same **SMSSF ID** or the same **RDKit SMILES** are grouped together. The grouping operation assigns them the same **Molecule ID** and different **Duplicate ID**s. This allows us to compute aggregate % inhibition scores by grouping on **Molecule ID**.

In addition to grouping replicate measurements, we also ensure that a single experimental measurement is not erroneously recorded twice. Entries in the combined dataset should not match in the following columns: **SMSSF ID**, **Plate Name**, **Plate Well**, **Run Date**, **PriA-SSB AS % inhibition**. If two entries match on these columns, the same data have been copied so one entry is removed.

A.4 Combined Dataset Preprocessing Steps

The preprocessing steps for the combined dataset are as follows:

1. Read in the merged five individual measurement files.

2. Remove 140 outliers with % inhibition ≤ -100.0 based on inspection of the % inhibition distribution.

3. Remove rows with missing values. Some molecules had **SMSSF ID** present, but other entries like **CDD SMILES**, **Plate Name**, etc. missing.

4. Define unique identifiers for each row to [**SMSSF ID**, **Plate Name**, **Plate Well**, **Run Date**, **PriA-SSB AS % inhibition**]. Assert that there are no duplicates on the uniqueness columns.

5. Add **RDKit SMILES** and fingerprints. Remove salts using RDKit's **SaltRemover** and **Salts.txt** (`https://github.com/rdkit/rdkit/blob/master/Data/Salts.txt`).

6. Add **Molecule ID** and **Duplicate ID** placeholders. Group molecules that have the same **SMSSF ID** or **RDKit SMILES** giving them the same **Molecule ID** and increasing **Duplicate ID**.

7. Generate binary labels **PriA-SSB AS Activity** according to the binarization rules below.

8. Save the combined dataset containing 441,900 molecules.

A.5 Binary Activity Rules

Molecules can have up to four % inhibition score measurements (same **Molecule ID** but different **Duplicate ID**). We defined the following rules for creating binary activity labels for training classifiers:

1. The median % inhibition value over all **primary** screens of the molecule is ≥ 35%.

2. If one or more retest screens were conducted, the median % inhibition value over all **retest** screens of the molecule is ≥ 35%.

3. The molecule does not match a PAINS filter per RDKit's **FilterCatalog**.

These rules are applied to all molecules individually by grouping using the **Molecule ID**. The binary activity label is assigned to all instances of the molecule identified by **Molecule ID**.

A.6 Generating the Training Dataset

The combined dataset can have many % inhibition readings for a single molecule. The training dataset will contain a single entry for each unique molecule identified by its **Molecule ID**. It is generated as follows:

1. Read in the combined dataset.

2. Remove retests, leaving the primary screens. Recall that the binary activity is still recorded in the primary entries.

3. Standardize **Library ID**s to support stratifying by library.

4. Group by **Molecule ID** and compute the median % inhibition for the primary screens in the **PriA-SSB AS % inhibition (Primary Median)** column.

5. Save this as the training dataset.

Each entry in the training dataset has a unique **Molecule ID** and is thus regarded as a unique molecule. The method to generate folds for cross validation starts by grouping molecules based on the **Library ID** column. The next step performs stratified sampling on each **Library ID** value, grouping it into 10 folds based on the binary activity labels. This ensures that each library is well represented in each fold and the activity ratios are similar. For example, if LC1 has 1,000 total molecules with 10 actives, then each of the 10 folds will have 1 LC1 active and 99 LC1 inactives.

A.7 Supervised Learning Model Hyperparameters

Here we describe the best hyperparameters combinations for the RF-C, XGB-C, XGB-R, NN-C, and NN-R models. We performed grid searches over the hyperparameter values in the tables referenced below. Each combination of hyperparameters in the grid search was assigned a numeric ID. The mapping from numeric IDs to hyperparameter combinations is available in our GitHub repository.

For each model, we report the top-performing hyperparameter IDs sorted by performance. Some training jobs took over a week to complete. We recorded results for all the jobs that terminated within a week and randomly picked additional long jobs to run to termination. The remaining long jobs are reported below as missing hyperparameter IDs because they did not generate results.

Table S15 shows the 216 RF-C hyperparameters. Top 20 (with ties) hyperparameter IDs: 139, 69, 111, 210, 148, 212, 28, 61, 124, 130, 131, 141, 14, 38, 165, 65, 123, 94, 3, 88, 72. Missing hyperparameter IDs: 5, 6, 19, 23, 24, 26, 27, 29, 37, 47, 52, 64, 71, 75, 91, 101, 113, 114, 126, 133, 136, 137, 145, 153, 156, 158, 160, 167, 168, 179, 187, 189, 191, 193, 201, 208.

Table S16 shows the XGB-C hyperparameters. There are 1,866,240 hyperparameter combinations from which we randomly selected 1,000. Top 20 (with ties) hyperparameter IDs: 140, 967, 807, 263, 694, 440, 47, 116, 792, 663, 32, 564, 950, 84, 364, 605, 431, 55, 388, 960, 735. No missing hyperparameter IDs.

Table S17 shows the XGB-R hyperparameters. There are 829,440 hyperparameter combinations from which we randomly selected 1,000. Top 20 (with ties) hyperparameter IDs are: 187, 880, 514, 605, 754, 6, 321, 753, 294, 718, 280, 214, 545, 507, 81, 65, 440, 409, 586, 721, 911. Missing hyperparameter IDs: 56, 120, 434, 589, 708, 874.

Table S18 shows the 432 NN-C hyperparameters. Top 20 hyperparameter IDs: 356, 88, 180, 5, 416, 183, 47, 124, 93, 385, 54, 423, 363, 370, 215, 415, 29, 132, 254, 404. Missing hyperparameter IDs: 7, 35, 48, 69, 73, 79, 91, 92, 95, 97, 113, 120, 122, 126, 137, 138, 142, 143, 166, 225, 231, 234, 237, 239, 251, 261, 275, 281, 284, 294, 297, 301, 303, 314, 317, 320,

336, 338, 340, 341, 342, 346, 368, 369, 372, 376, 378, 380, 382, 384, 389, 391, 395, 398, 399, 403, 420, 429, 431.

Table S19 shows the 432 NN-R hyperparameters. Top 20 hyperparameter IDs: 47, 199, 175, 27, 178, 20, 114, 106, 157, 270, 123, 191, 323, 369, 265, 17, 45, 115, 108, 67. Missing hyperparameter IDs: 7, 15, 26, 69, 92, 93, 137, 138, 166, 231, 234, 237, 281, 297, 303, 336, 338, 341, 346, 380, 398, 403.

A.8 Ensemble Model Training

The Model-Based Ensemble and Max Vote Ensemble models are introduced in the model selection stage. Both ensemble models make use of N base model predictions as input and produce a single activity probability prediction as output. The Max Vote Ensemble uses a heuristic strategy that takes the maximum of the base model predictions. On the other hand, the Model-Based Ensemble trains an XGB model with the base model predictions as input.

The steps for training the ensemble models (depicted in Figure S3) are as follows:

- **Step 1:** The base models are trained on folds 0-8 with fold 8 being used as the validation fold. The RF models do not require a validation set, so they do not make use of fold 8.

- **Step 2:** The trained base models from step 1 are then used to generate predictions for folds 0-7 and fold 9.

- **Step 3:** All N base model predictions from step 2 for folds 0-7 are concatenated (stacked) to construct ensemble features. The Model-Based Ensemble is then trained with these ensemble features as input and the true labels as output for folds 0-7. For the Max Vote Ensemble, no training is needed as the output is the maximum of the ensemble features.

- **Step 4:** All N base model predictions from step 2 for fold 9 are concatenated. The trained ensemble from step 3 is then used to generate the final fold 9 prediction with the concatenated features as input.

There are several ways in which our ensembling approach could be improved. In a typical stacking ensemble,[76] the ensemble is trained on predictions that the base models did not train on. If the training set is denoted as A, then one common stacking method is to do K-fold cross validation with the base models and then use the stacked K held-out fold predictions to train the ensemble. Another strategy is to split A into two subsets, one for training the base models, and one for generating base model predictions to train the ensemble. However, we train the base models on a dataset A, generate predictions on A, and train the ensemble on these predictions. This can introduce a bias in a parameter-based ensemble like Model-Based Ensemble towards favoring base models that have a better fit on A. The Max Vote Ensemble does not have this potential bias because it is parameter free.

In addition, we could have used a more flexible and thorough strategy to select the base model mixtures and excluded ensemble configurations with a single base model (Table S20). In retrospect, we observed that including regression models in the ensembles hurt performance (Figures S1 and S2). Furthermore, the regression base model outputs were not scaled, which was likely detrimental to the Max Vote Ensemble when combining classification and regression models. The Model-Based Ensemble is less affected by the scale of the regression model outputs because its XGB model can account for different score distributions provided by each base model.

A.9 Enamine REAL Dose-Response Curves

We generated three sets of dose-response curves: eight-point curves with four replicates for all 68 Enamine compounds, 16-point curves with eight replicates for 10 compounds, and another set of 16-point curves with six replicates for 10 compounds. The eight-point dose-response curves started with an initial concentration of 66 µM and progressively divided the

concentration in half until reaching 515.6 nM. Both 16-point dose-response curves started with an initial concentration of 100 µM, then 66.7 µM, and then progressively divided the concentration in half until reaching 4.2 nM. All dose-response screens followed the same protocol used for the MLPCN library. The dose-response curve images are available in our Zenodo dataset.

We generated the 16-point dose-response curves because we were unable to reliably establish IC50 values for some compounds in the eight-point screen. The minimum concentrations tested were not low enough. When reporting IC50 values and bounds in Table 4 we used the screening data from the highest-quality dose-response curve. The second set of 16-point curves were the highest quality because they had 16 concentrations and were unaffected by any spatial plate effects. The first set of 16-point curves were the next highest quality because they also had 16 concentrations and the most replicates. However, some of the readings at the lowest concentration in these curves were skewed due to assay spatial effects at the plate edge. The eight-point curves were used only if the compound was not tested in a 16-point curve. Some of the replicates at the lowest concentration in the eight-point curves were also affected by the spatial effects.

The Collaborative Drug Discovery Vault software[83] modifies the original dose-response classification criteria.[82] It defines curve class 1 as a "complete curve, showing multiple points near both asymptotes" and curve class 2 as a "partial curve, showing multiple points near only one asymptote." The 1.2 and 2.2 subclasses indicate that the curve fit has $R^2 > 0.9$ and maximum activity $\leq 80\%$.

B Supplementary Results

B.1 Base Model versus Ensemble Model Actives

In the model selection stage, fold 9 was used for performance comparison among models. The motivation for using ensembles was to pool the predictions of various models and thus

promote diversity in the top predicted compounds. We compared the prediction overlap between the ensembles and the best base models in the model selection stage (Table 1). Specifically, for each top base model, we compared the top 1% predicted compounds from fold 9 against the top 1% predicted by the ensembles (Tables S2-S6). This examines the similarity and difference in the actives retrieved by the top base models and the ensembles.

These predictions for the Model-Based Ensemble were regenerated using a newer version of Keras. However, the performance of the regenerated ensemble and the ensemble referred to in Table 1 differs by no more than 10^{-3}.

B.2 AMS Compound Activity Level versus Cluster Membership

The 1,024 AMS compounds were divided into three activity level categories based on their % inhibition values in the two replicates:

- **Weak Actives:** Compounds with % inhibition in $[35.0, 50.0)$ in both replicates. The value 35.0 is close to the 40th percentile in replicate 1.
- **Normal Actives:** Compounds with % inhibition between $[50.0, 72.0)$ in both replicates. The value 50.0 is the threshold used to define hits.
- **Strong Actives:** Compounds with % inhibition ≥ 72.0 in both replicates. The value 72.0 is close to the 75th percentile in replicate 1.

Table S8 summarizes the AMS compound counts by activity level. Note that the sum of normal and strong actives is equal to the total hits. A Jupyter notebook in our GitHub repository shows the cluster membership versus activity level counts. We performed Fisher's exact test using the stats package in R with the following arguments: `workspace=2e8`, `hybrid=FALSE`, `alternative="two.sided"`, `conf.level=0.95`, `simulate.p.value=TRUE`.

B.3 Scaling to a Billion Compounds with Enamine REAL

Using the top RF-C model from the model selection stage trained on the training set, we scored the 1,077,562,987 Enamine REAL compounds. A total of 18 jobs, each processing about 60.3 million compounds, were processed on 18 independent compute nodes with a single CPU core, 6GB RAM, and 30GB disk space allocated to each job. Table S11 summarizes the processing time in hours and number of compounds per node. The mean time was 53.21 hours with a 6.40 hour standard deviation. Because each compound is scored independently, we could easily reduce the total wall time needed to score over a billion compounds by distributing the compounds across hundreds or thousands of compute nodes. The Similarity Baseline ran under similar conditions and had a mean time of 19.48 hours with a 1.68 hour standard deviation.

C Supplementary Figures

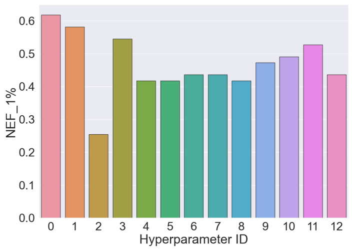

(a) $NEF_{1\%}$

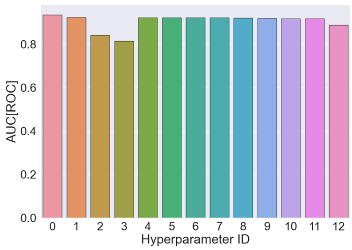

(b) AUC[ROC]

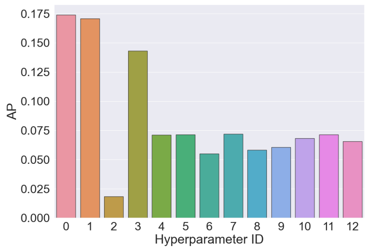

(c) AP

Figure S1: Model-Based Ensemble results on fold 9. Table S20 lists the base models associated with each ensemble hyperparameter ID.

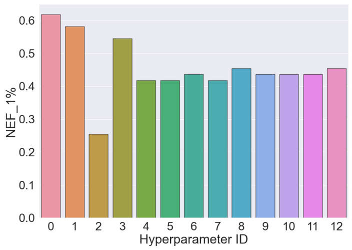

(a) $NEF_{1\%}$

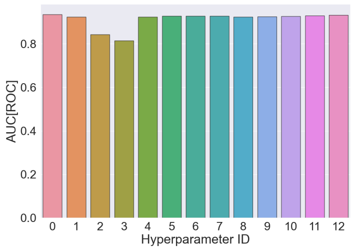

(b) AUC[ROC]

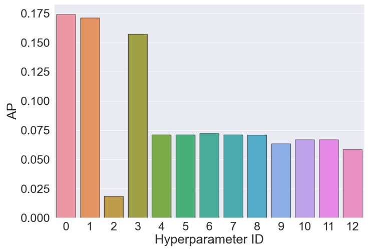

(c) AP

Figure S2: Max Vote Ensemble results on fold 9. Table S20 lists the base models associated with each ensemble hyperparameter ID.

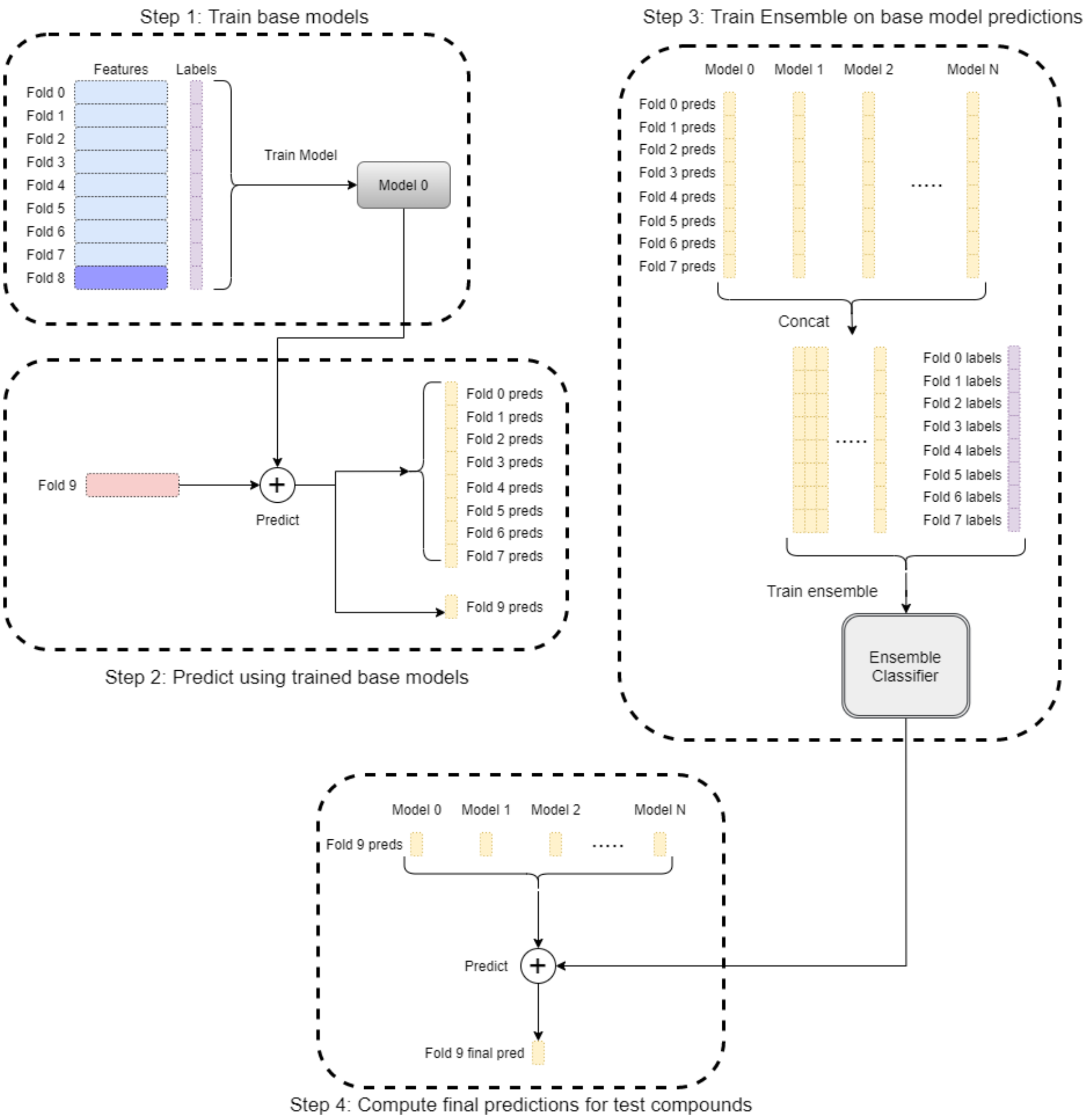

Figure S3: Illustration of ensemble training steps in the context of the model selection stage. Step 1 trains base models on folds 0-8 with fold 8 being used as the validation fold. Step 2 generates predictions on folds 0-7 and fold 9. Step 3 trains the ensemble classifier on base model predictions for folds 0-7. Step 4 uses the trained ensemble to generate final predictions on fold 9.

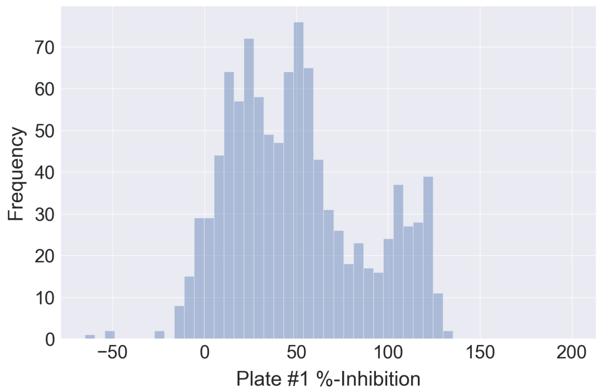

(a) Replicate 1

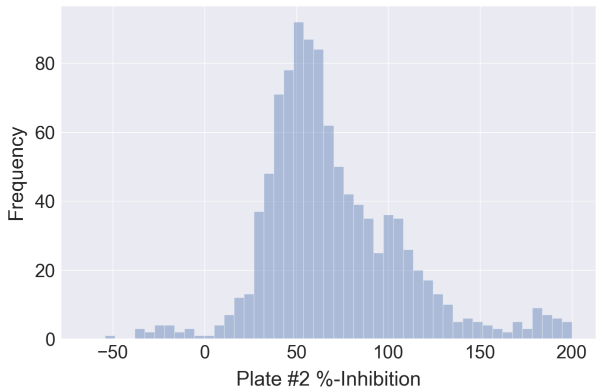

(b) Replicate 2

Figure S4: % inhibition distributions of the 1,024 AMS compounds for both replicates.

D Supplementary Tables

Table S1: Cross validation (CV) training, validation, and test fold splits for each run ID. A total of four runs were done for each hyperparameter setting in the hyperparameter tuning stage. Ensemble models followed a different splitting scheme.

CV run ID	Validation fold	Test fold	Training folds
0	0	1	2, 3, 4, 5, 6, 7
1	2	3	0, 1, 4, 5, 6, 7
2	4	5	0, 1, 2, 3, 6, 7
3	6	7	0, 1, 2, 3, 4, 5

Table S2: Top RF-C model versus the ensembles. Set difference of actives when filtering for the top 427 predictions on fold 9 in the model selection stage.

Model	Ensemble	Ensemble \ Model	Model \ Ensemble
RF-C	Model-Based #0	0	0
RF-C	Model-Based #1	1	3
RF-C	Model-Based #2	2	22
RF-C	Model-Based #3	0	4
RF-C	Model-Based #4	2	13
RF-C	Model-Based #5	2	13
RF-C	Model-Based #6	3	13
RF-C	Model-Based #7	2	12
RF-C	Model-Based #8	2	13
RF-C	Model-Based #9	3	11
RF-C	Model-Based #10	2	8
RF-C	Model-Based #11	2	7
RF-C	Model-Based #12	1	11
RF-C	Max Vote #0	0	0
RF-C	Max Vote #1	1	3
RF-C	Max Vote #2	2	22
RF-C	Max Vote #3	0	4
RF-C	Max Vote #4	2	13
RF-C	Max Vote #5	2	13
RF-C	Max Vote #6	2	12
RF-C	Max Vote #7	2	13
RF-C	Max Vote #8	2	11
RF-C	Max Vote #9	2	12
RF-C	Max Vote #10	2	12
RF-C	Max Vote #11	2	12
RF-C	Max Vote #12	1	10

Table S3: Top XGB-C model versus the ensembles. Set difference of actives when filtering for the top 427 predictions on fold 9 in the model selection stage.

Model	Ensemble	Ensemble \ Model	Model \ Ensemble
XGB-C	Model-Based #0	3	1
XGB-C	Model-Based #1	0	0
XGB-C	Model-Based #2	2	20
XGB-C	Model-Based #3	2	4
XGB-C	Model-Based #4	2	11
XGB-C	Model-Based #5	2	11
XGB-C	Model-Based #6	4	12
XGB-C	Model-Based #7	3	11
XGB-C	Model-Based #8	3	12
XGB-C	Model-Based #9	4	10
XGB-C	Model-Based #10	4	8
XGB-C	Model-Based #11	4	7
XGB-C	Model-Based #12	3	11
XGB-C	Max Vote #0	3	1
XGB-C	Max Vote #1	0	0
XGB-C	Max Vote #2	2	20
XGB-C	Max Vote #3	2	4
XGB-C	Max Vote #4	2	11
XGB-C	Max Vote #5	2	11
XGB-C	Max Vote #6	3	11
XGB-C	Max Vote #7	2	11
XGB-C	Max Vote #8	4	11
XGB-C	Max Vote #9	3	11
XGB-C	Max Vote #10	3	11
XGB-C	Max Vote #11	3	11
XGB-C	Max Vote #12	2	9

Table S4: Top XGB-R model versus the ensembles. Set difference of actives when filtering for the top 427 predictions on fold 9 in the model selection stage.

Model	Ensemble	Ensemble \ Model	Model \ Ensemble
XGB-R	Model-Based #0	22	2
XGB-R	Model-Based #1	20	2
XGB-R	Model-Based #2	0	0
XGB-R	Model-Based #3	18	2
XGB-R	Model-Based #4	14	5
XGB-R	Model-Based #5	14	5
XGB-R	Model-Based #6	13	3
XGB-R	Model-Based #7	15	5
XGB-R	Model-Based #8	12	3
XGB-R	Model-Based #9	15	3
XGB-R	Model-Based #10	18	4
XGB-R	Model-Based #11	19	4
XGB-R	Model-Based #12	14	4
XGB-R	Max Vote #0	22	2
XGB-R	Max Vote #1	20	2
XGB-R	Max Vote #2	0	0
XGB-R	Max Vote #3	18	2
XGB-R	Max Vote #4	14	5
XGB-R	Max Vote #5	14	5
XGB-R	Max Vote #6	15	5
XGB-R	Max Vote #7	14	5
XGB-R	Max Vote #8	16	5
XGB-R	Max Vote #9	15	5
XGB-R	Max Vote #10	15	5
XGB-R	Max Vote #11	15	5
XGB-R	Max Vote #12	15	4

Table S5: Top NN-C model versus the ensembles. Set difference of actives when filtering for the top 427 predictions on fold 9 in the model selection stage.

Model	Ensemble	Ensemble \ Model	Model \ Ensemble
NN-C	Model-Based #0	4	0
NN-C	Model-Based #1	4	2
NN-C	Model-Based #2	2	18
NN-C	Model-Based #3	0	0
NN-C	Model-Based #4	2	9
NN-C	Model-Based #5	2	9
NN-C	Model-Based #6	3	9
NN-C	Model-Based #7	2	8
NN-C	Model-Based #8	2	9
NN-C	Model-Based #9	3	7
NN-C	Model-Based #10	2	4
NN-C	Model-Based #11	2	3
NN-C	Model-Based #12	1	7
NN-C	Max Vote #0	4	0
NN-C	Max Vote #1	4	2
NN-C	Max Vote #2	2	18
NN-C	Max Vote #3	0	0
NN-C	Max Vote #4	2	9
NN-C	Max Vote #5	2	9
NN-C	Max Vote #6	2	8
NN-C	Max Vote #7	2	9
NN-C	Max Vote #8	2	7
NN-C	Max Vote #9	2	8
NN-C	Max Vote #10	2	8
NN-C	Max Vote #11	2	8
NN-C	Max Vote #12	1	6

Table S6: Top NN-R model versus the ensembles. Set difference of actives when filtering for the top 427 predictions on fold 9 in the model selection stage.

Model	Ensemble	Ensemble \ Model	Model \ Ensemble
NN-R	Model-Based #0	13	2
NN-R	Model-Based #1	11	2
NN-R	Model-Based #2	5	14
NN-R	Model-Based #3	9	2
NN-R	Model-Based #4	0	0
NN-R	Model-Based #5	0	0
NN-R	Model-Based #6	3	2
NN-R	Model-Based #7	1	0
NN-R	Model-Based #8	3	3
NN-R	Model-Based #9	4	1
NN-R	Model-Based #10	5	0
NN-R	Model-Based #11	6	0
NN-R	Model-Based #12	4	3
NN-R	Max Vote #0	13	2
NN-R	Max Vote #1	11	2
NN-R	Max Vote #2	5	14
NN-R	Max Vote #3	9	2
NN-R	Max Vote #4	0	0
NN-R	Max Vote #5	0	0
NN-R	Max Vote #6	1	0
NN-R	Max Vote #7	0	0
NN-R	Max Vote #8	2	0
NN-R	Max Vote #9	1	0
NN-R	Max Vote #10	2	1
NN-R	Max Vote #11	2	1
NN-R	Max Vote #12	3	1

Table S7: Summary of cluster hit metrics for the 1,024 AMS compounds. Unique cluster (U. Cl.) hits denotes the number of clusters containing at least one hit. Novel cluster (Nov. Cl.) hits denotes the number of clusters with AMS hits but no training set actives. Taylor-Butina (TB) clustering with the specified distance thresholds is used to define clusters.

Selector	U. Cl. Hits (TB 0.2)	Nov. Cl. Hits (TB 0.2)	U. Cl. Hits (TB 0.3)	Nov. Cl. Hits (TB 0.3)	U. Cl. Hits (TB 0.4)	Nov. Cl. Hits (TB 0.4)
RF-C or Baseline	351	304	242	138	169	72
RF-C	295	258	217	132	142	61
Baseline	200	153	124	21	115	30
RF-C and Baseline	144	107	99	15	88	19
RF-C but not Baseline	151	151	130	120	72	44
Baseline but not RF-C	63	48	43	7	40	13

Table S8: The 1,024 AMS compounds grouped by activity level. Only Strong Actives and Normal Actives were considered to be hits in all primary analyses. Weak Actives fell below the % inhibition threshold.

Selector	Count	Hits	Strong Actives	Normal Actives	Weak Actives
RF-C or Baseline	1,024	412	221	191	178
RF-C	701	337	208	129	110
Baseline	705	256	111	145	146
RF-C and Baseline	382	181	98	83	78
RF-C but not Baseline	319	156	110	46	32
Baseline but not RF-C	323	75	13	62	68

Table S9: Illustration of the five AMS hits selected by RF-C that are the farthest from their nearest training set active. Tanimoto distance is 1 - Tanimoto similarity.

AMS Hit	Closest Training Active	Similarity Map	Tanimoto Distance
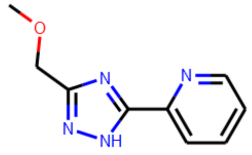	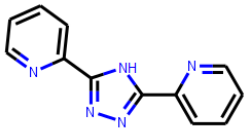	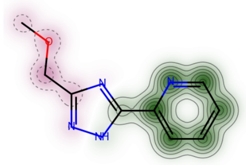	0.57
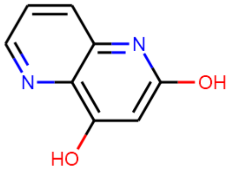	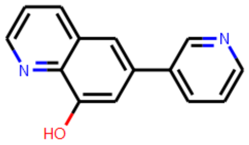	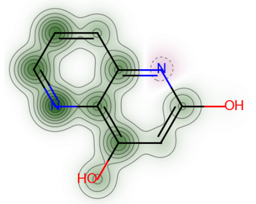	0.56
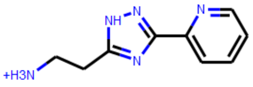	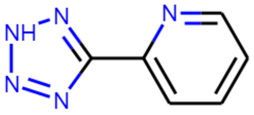	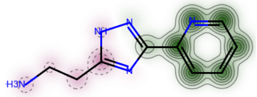	0.55
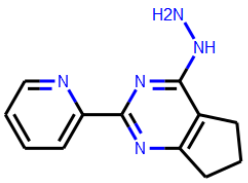	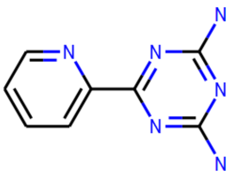	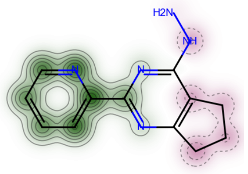	0.55
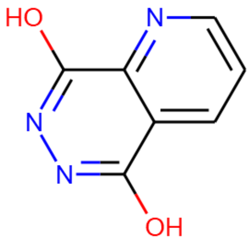	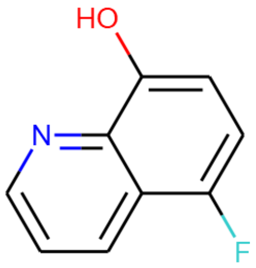	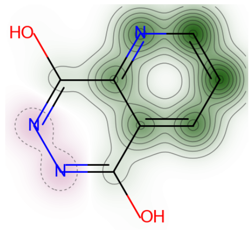	0.53

Table S10: Illustration of the five AMS hits selected by the Similarity Baseline that are the farthest from their nearest training set active. Tanimoto distance is 1 - Tanimoto similarity.

AMS Hit	Closest Training Active	Similarity Map	Tanimoto Distance
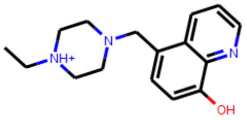	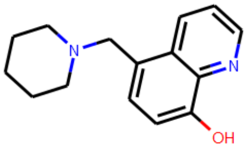	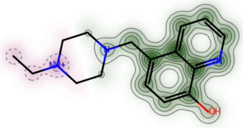	0.32
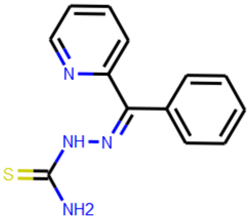	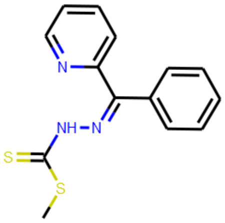	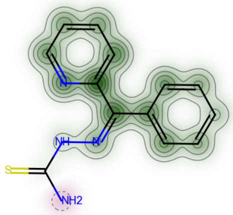	0.32
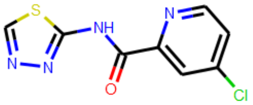	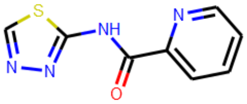	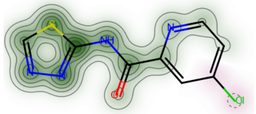	0.32
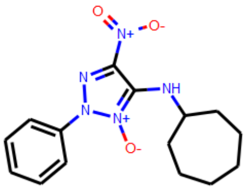	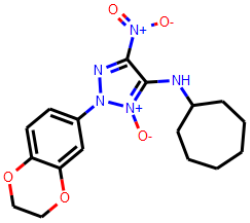	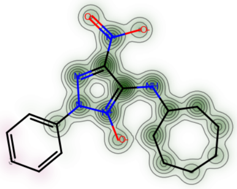	0.31
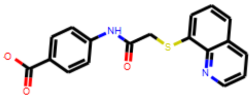	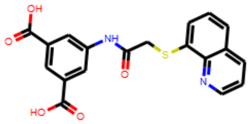	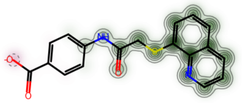	0.31

Table S11: Timing estimates for RF-C prediction on the Enamine REAL library consisting of 1,077,562,987 compounds. The library processing was split among 18 compute nodes.

Part	Hours	# compounds
0	50.83	60262531
1	63.71	60262531
2	46.62	60262531
3	45.81	60262531
4	63.43	60262531
5	63.70	60262531
6	51.81	60262531
7	64.35	60262531
8	49.12	60262531
9	49.86	60262531
10	52.74	60262531
11	46.51	60262530
12	54.52	60263531
13	49.47	60263531
14	47.12	60263531
15	55.65	60263531
16	55.88	60263531
17	46.63	53094961
Mean	53.21	59864610
Std	6.40	1641881

Table S12: The overlap of the top K Enamine REAL compounds selected by the RF-C and Similarity Baseline models. Both models prioritized compounds from the entire Enamine REAL dataset. Then, the top K predicted compounds from both models were compared for overlap.

Top K	Overlap count	Overlap %
50	17	34.00%
100	25	25.00%
250	65	26.00%
500	120	24.00%
1000	220	22.00%
5000	936	18.72%
10000	2013	20.13%
20000	4414	22.07%
30000	6652	22.17%
40000	8728	21.82%
50000	10703	21.41%
60000	12534	20.89%
70000	14301	20.43%
80000	16004	20.00%
90000	17739	19.71%
100000	19363	19.36%

Table S13: The 40 inactive Enamine REAL compounds, which include some compounds that were called as initial hits but not confirmed in the full dose-response curves. The most similar known active compound from the training set or AMS compounds is shown along with its Tanimoto distance (which is 1 - Tanimoto similarity) to the Enamine inactive. The similarity maps from RDKit show similar substructures in green and dissimilar substructures in red. Inactives that match AlphaScreen frequent hitters filters[33] are denoted with a checkmark.

Enamine Inactive	Similarity Map Nearest Active Tanimoto Distance	Similarity Map Nearest Inactive Tanimoto Distance	Matches AS Freq. Hitter
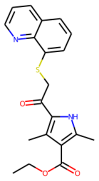 Z71173293	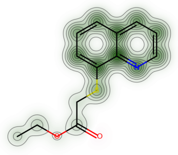 0.39	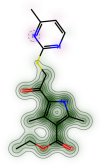 0.37	
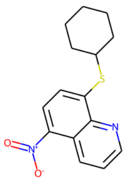 PV-002161956828	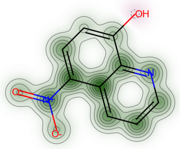 0.44	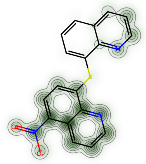 0.43	
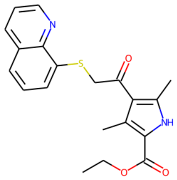 Z225905608	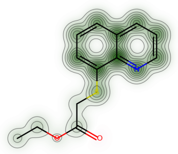 0.39	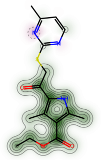 0.48	
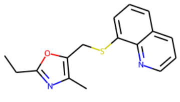 Z2273856428	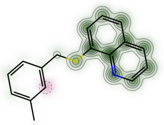 0.47	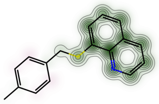 0.45	
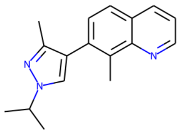 Z2618185084	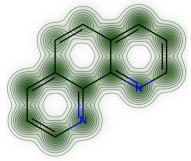 0.57	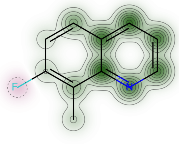 0.56	

Continued on next page

Table S13 – Continued from previous page.

Enamine Inactive	Similarity Map Nearest Active Tanimoto Distance	Similarity Map Nearest Inactive Tanimoto Distance	Matches AS Freq. Hitter
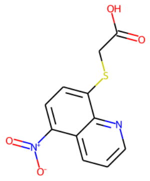 Z285779920	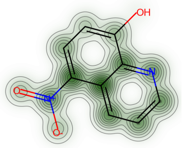 0.36	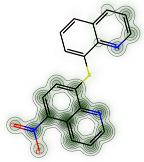 0.42	
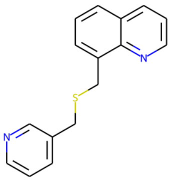 Z2146195633	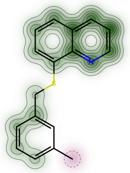 0.56	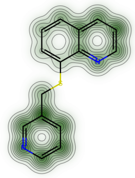 0.32	
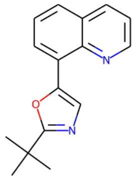 Z2998111045	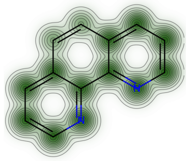 0.57	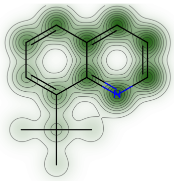 0.49	
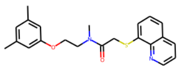 PV-001915624464	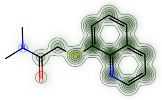 0.38	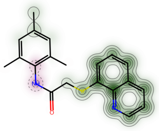 0.47	
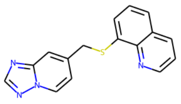 PV-002140113355	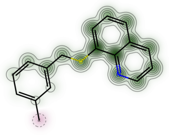 0.43	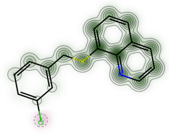 0.43	
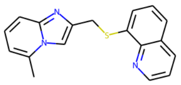 Z56788250	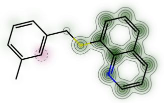 0.47	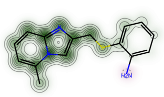 0.33	

Continued on next page

Table S13 – Continued from previous page.

Enamine Inactive	Similarity Map Nearest Active Tanimoto Distance	Similarity Map Nearest Inactive Tanimoto Distance	Matches AS Freq. Hitter
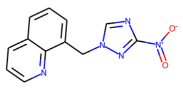 Z666250890	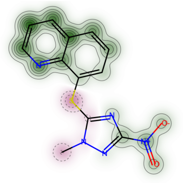 0.51	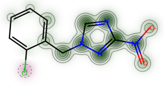 0.4	
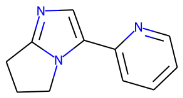 Z3347084877	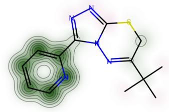 0.62	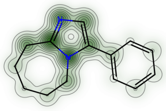 0.44	✓
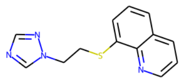 Z1116571364	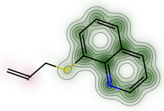 0.49	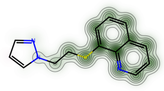 0.3	
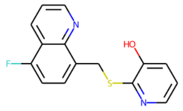 PV-002126332674	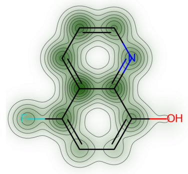 0.44	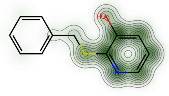 0.5	
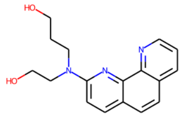 Z3557694708	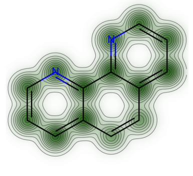 0.54	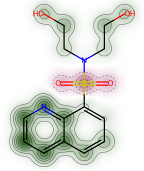 0.56	
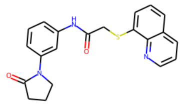 Z202503096	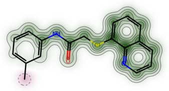 0.36	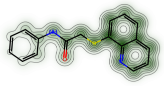 0.36	

Continued on next page

Table S13 – Continued from previous page.

Enamine Inactive	Similarity Map Nearest Active Tanimoto Distance	Similarity Map Nearest Inactive Tanimoto Distance	Matches AS Freq. Hitter
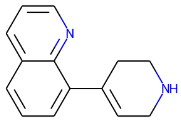 Z3238971184	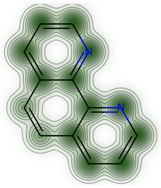 0.55	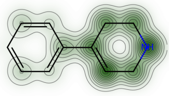 0.57	
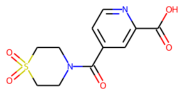 Z1443864314	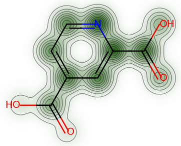 0.43	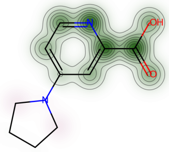 0.54	
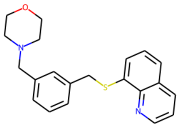 PV-001915624917	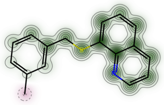 0.42	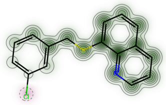 0.42	
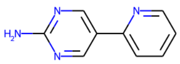 Z1255380138	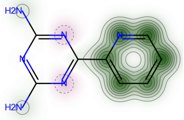 0.47	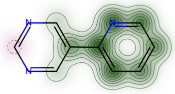 0.39	
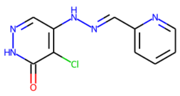 Z114886052	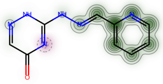 0.41	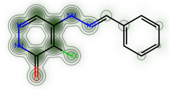 0.31	
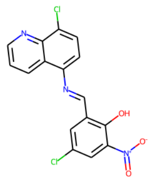 Z314947084	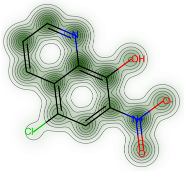 0.42	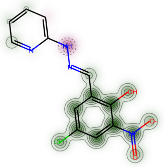 0.45	

Continued on next page

Table S13 – Continued from previous page.

Enamine Inactive	Similarity Map Nearest Active Tanimoto Distance	Similarity Map Nearest Inactive Tanimoto Distance	Matches AS Freq. Hitter
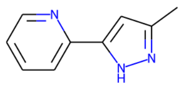 Z3225506284	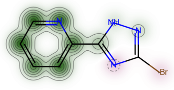 0.54	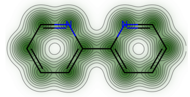 0.5	
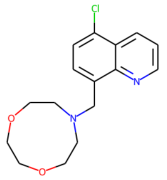 Z3652859018	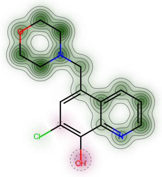 0.35	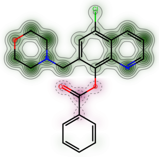 0.43	
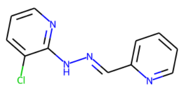 Z734817070	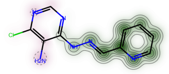 0.4	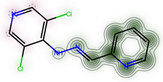 0.38	
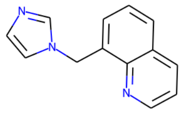 Z241926076	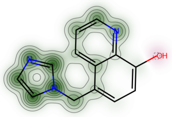 0.4	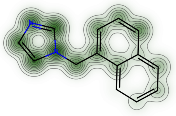 0.35	
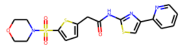 Z29467739	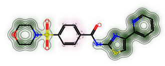 0.36	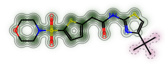 0.31	✓
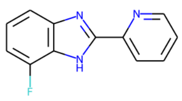 Z666485712	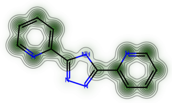 0.53	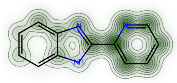 0.41	✓

Continued on next page

Table S13 – Continued from previous page.

Enamine Inactive	Similarity Map Nearest Active Tanimoto Distance	Similarity Map Nearest Inactive Tanimoto Distance	Matches AS Freq. Hitter
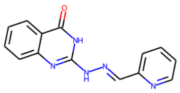 Z1407899729	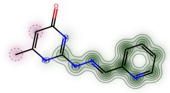 0.4	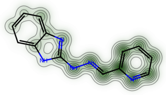 0.31	
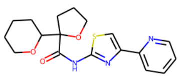 Z3075781550	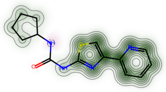 0.45	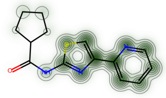 0.43	✓
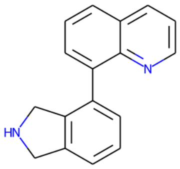 Z3484513235	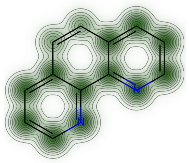 0.55	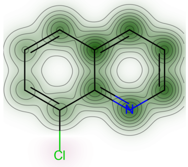 0.57	
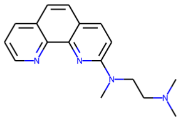 Z3557781825	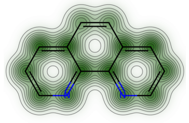 0.5	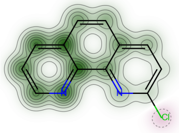 0.55	
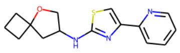 Z3295148399	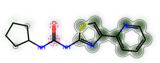 0.51	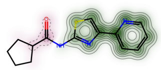 0.52	✓
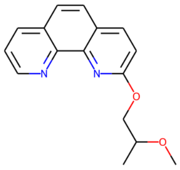 Z3634004206	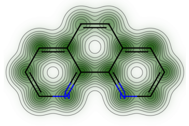 0.53	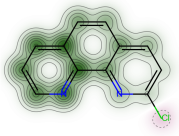 0.57	

Continued on next page

Table S13 – Continued from previous page.

Enamine Inactive	Similarity Map Nearest Active Tanimoto Distance	Similarity Map Nearest Inactive Tanimoto Distance	Matches AS Freq. Hitter
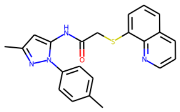 Z71174619	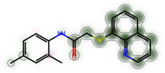 0.37	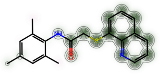 0.35	
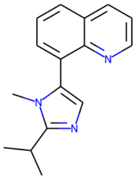 Z2739840324	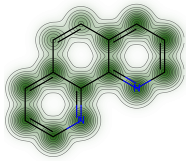 0.58	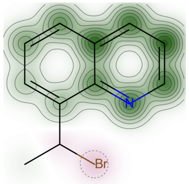 0.58	
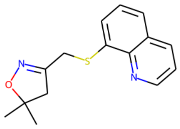 Z3638513333	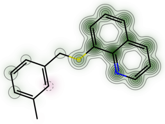 0.47	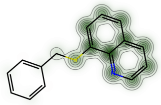 0.48	
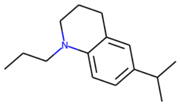 PV-001918010086	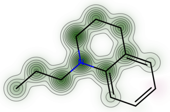 0.41	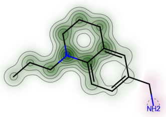 0.37	
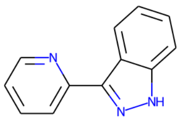 Z3242476057	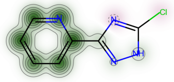 0.56	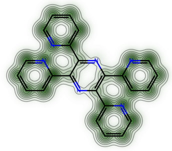 0.48	✓

Table S14: Summary of fold information denoting the number of compounds, number of clusters, and number of clusters not present in other folds. Compounds were clustered using Taylor-Butina at the 0.4 distance threshold.

Fold ID	# Compounds	# Clusters	# Clusters not in other folds
0	42729	26753	3377
1	42730	26734	3392
2	42737	26696	3276
3	42734	26709	3326
4	42730	26708	3352
5	42729	26772	3342
6	42730	26746	3397
7	42727	26717	3345
8	42727	26783	3442
9	42727	26667	3386
All folds	427300	88324	33635

Table S15: 216 RF-C hyperparameter combinations.

Hyperparameter	Hyperparameter values
number of estimators	50; 250; 1000; 4000; 8000; 16000
max features	None; Sqrt; Log2
min sample leaf	1; 10; 100; 1000
class weight	None; balanced subsample, balanced

Table S16: 1,866,240 XGB-C hyperparameter combinations from which we randomly select 1,000.

Hyperparameter	Hyperparameter values
max depth	5; 10; 50; 100
learning rate	1; 3e-1; 1e-1; 3e-2
number of estimators	30; 100; 300; 1000; 3000
subsample	1; 0.5
colsample bylevel	1; 0.5; 0.2
colsample bytree	1; 0.5; 0.2
min child weight	1; 5; 20
reg alpha	0; 0.3
reg lambda	1; 0.7
scale pos weight	1; 0.1; 0.001
max delta step	0; 1; 3
eval metric	AVG[PR]; AUC[ROC]
eval round	100; 300; 1000
booster	gbtree; gblinear

Table S17: 829,440 XGB-R hyperparameter combinations from which we randomly select 1,000.

Hyperparameter	Hyperparameter values
max depth	5; 10; 50; 100
learning rate	1; 3e-1; 1e-1; 3e-2
number of estimators	30; 100; 300; 1000; 3000
subsample	1; 0.5
colsample bylevel	1; 0.5; 0.2
colsample bytree	1; 0.5; 0.2
min child weight	1; 5; 20
reg alpha	0; 0.3
reg lambda	1; 0.7
scale pos weight	1; 0.3; 0.1; 0.003
eval metric	AVG[PR]; AUC[ROC]
eval round	100; 300; 1000
booster	gbtree; gblinear

Table S18: 432 NN-C hyperparameter combinations.

Hyperparameter	Hyperparameter values
learning rate	0.00003; 0.0001; 0.003
weighted schema	no weight; weighted sample
epoch patience	epoch size: 200, patience: 50;<br>epoch size: 1000, patience: 200
early stopping	AUC[ROC]; AVG[PR]
dropout	0.1, 0.25
layers	500(ReLU), 200(Sigmoid), 1(Sigmoid);<br>1000(ReLU), 500(Sigmoid), 1(Sigmoid);<br>2000(ReLU), 2000(Sigmoid), 1(Sigmoid);<br>2000(ReLU), 2000(ReLU), 1(Sigmoid);<br>2000(Sigmoid), 2000(Sigmoid), 1(Sigmoid);<br>2000(ReLU), 4000(Sigmoid), 2000(Sigmoid), 1(Sigmoid);<br>2000(ReLU), 1000(Sigmoid), 1(Sigmoid);<br>2000(ReLU), 4000(Sigmoid), 1000(Sigmoid), 1(Sigmoid);<br>2000(ReLU), 4000(Sigmoid), 8000(Sigmoid), 1000(Sigmoid), 1(Sigmoid)

Table S19: 432 NN-R hyperparameter combinations.

Hyperparameter	Hyperparameter values
learning rate	0.00003; 0.0001; 0.003
weighted schema	no weight; weighted sample
epoch patience	epoch size: 200, patience: 50;<br>epoch size: 1000, patience: 200
early stopping	AUC[ROC]; AVG[PR]
dropout	0.1, 0.25
layers	500(ReLU), 200(Sigmoid), 1(Linear);<br>1000(ReLU), 500(Sigmoid), 1(Linear);<br>2000(ReLU), 2000(Sigmoid), 1(Linear);<br>2000(ReLU), 2000(ReLU), 1(Linear);<br>2000(Sigmoid), 2000(Sigmoid), 1(Linear);<br>2000(ReLU), 4000(Sigmoid), 2000(Sigmoid), 1(Linear);<br>2000(ReLU), 1000(Sigmoid), 1(Linear);<br>2000(ReLU), 4000(Sigmoid), 1000(Sigmoid), 1(Linear);<br>2000(ReLU), 4000(Sigmoid), 8000(Sigmoid), 1000(Sigmoid), 1(Linear)

Table S20: The 13 different variants of the ensemble models. An ensemble model combines the top N models from each type of base machine learning model, where N is listed below.

ID	RF-C	XGB-C	XGB-R	NN-C	NN-R
0	1	0	0	0	0
1	0	1	0	0	0
2	0	0	1	0	0
3	0	0	0	1	0
4	0	0	0	0	1
5	1	1	1	1	1
6	2	2	2	2	2
7	2	2	1	2	1
8	5	5	3	5	3
9	10	10	5	10	5
10	5	5	10	5	10
11	20	20	10	20	10
12	10	10	20	10	20
